# The impact of World War II Army service on income and mobility in the 1960s by ethnoracial group

**DOI:** 10.1016/j.eeh.2025.101687

**Published:** 2025-04-17

**Authors:** Sergio E. Barrera, Andreas Ferrara, Price V. Fishback, Misty L. Heggeness

**Affiliations:** a Virginia Tech, Department of Economics, VA, USA; b University of Pittsburgh, Department of Economics, and NBER, PA, USA; c University of Arizona, Department of Economics, and NBER, AZ, USA; d University of Kansas, School of Public Affairs and Administration, Kansas, USA

**Keywords:** N12, J15, J71, Minority inequities, World War II, Veterans, Administrative records

## Abstract

We link the 1940 full-count Census to World War II enlistment records and 1969 administrative tax returns to study how WWII service in the Army and Army Airforce impacted the income and mobility of non-Hispanic White, Black, Hispanic, Asian, and Native American male Army veterans relative to their non-Army counterparts in 1969. The size of our data set provides enough power to shed new light on previously understudied groups, such as Hispanics, Asians, and Native Americans. In comparisons of Army veterans with non-Army men, Ordinary Least Squares estimates suggest that WWII Army veterans had higher incomes than non-Army men within the same group, and Army veterans were less likely to change counties between 1940 and 1969 than non-Army men within the non-Hispanic White, Black, and Hispanic groups. Worries about selection bias led us to estimate the effects with a fuzzy regression discontinuity design that compares men who were just too young to serve during World War II to men who were just old enough to serve. Those results showed that Army veterans had lower adjusted gross incomes than non-Army men within the non-Hispanic White, Black, and Asian groups, and slightly higher incomes within the Hispanic and Native American groups. The differences varied by type of income. Migration across county boundaries was lower for Army veterans than non-Army men among non-Hispanic Whites, Asians, and Native Americans, and there were only small differences among Blacks and Hispanics.

## 1. Introduction

World War II was one of the most devastating wars in history and it had profound impacts on the economies of belligerent countries. The United States, largely spared from wartime destruction at home, committed 16 million soldiers to fight the war – roughly 420,000 of whom died and another 600,000 who returned home wounded or disabled. The men and women who served in the military were often paid substantially below the earnings of similarly skilled civilians during the war. Soldiers may have gained certain skills from service during the war, but they also lost opportunities to continue their education or to build a career.

The federal government’s G.I. Bill sought to alleviate some of these disadvantages. It is therefore not obvious whether service in World War II had a positive, negative, or no effect on veterans’ labor market outcomes by the time they reached peak earning years. Prior work has shed light on this question (e.g. Angrist and Krueger, 1994; Turner and Bound, 2003) for Black and White veterans. Smaller but no less important groups, including Hispanic American, Asian American, and Native American veterans, have received less consideration (Browning et al., 1973), partly because the data previously available did not have enough observations to provide power for effective statistical analysis.

Our goal is to expand research on the impact of WWII service for a broader set of ethno-racial groups and to examine a wider range of outcomes than the literature has examined to date. The groups include Non-Hispanic Whites, Blacks, Hispanics, Asians, and Native Americans.^1^ The outcomes include wages and salaries, interest income, business/farm income, and overall income in 1969, when the men were in their prime earnings ages of 37–46. Further, we examine migration decisions and the characteristics of the areas where the men lived in 1969. We compare outcomes for Army veterans versus non-Army men within each group, as well as the Army effect for minority groups relative to Non-Hispanic White men, the largest group.

We match observations across several large-scale data sets that allow us to have a large enough sample size with the power to statistically analyze even smaller groups. We link almost 9 million Army and Army Air Force enlistment records hosted at the National Archives and Records Administration with the restricted-use full-count 1940 Census digitized by IPUMS (Ruggles et al., 2024a). We track male Army veterans and non-Army men over time by linking the merged census-and-enlistment data set to the universe of federal tax returns in 1969 via administrative identifiers based on the individuals’ social security numbers and other personally identifying information using the Census Bureau’s linkage process. The tax records provide information on a filer’s wage and salary income, their interest and dividend incomes, business and farm income and adjusted gross income, as well as their residence in 1969. The residence data allow us to measure changes in location between 1940 and 1969 and examine the characteristics of the Census tracts where they lived in 1969. Merging the WWII enlistment records with the 1940 Census and large-scale administrative tax records provides a sample of over 3 million men whom we observe in 1940 and again in 1969. The sample includes approximately 2.9 million Non-Hispanic White, 175,000 Black, 60,000 Hispanic, 9,000 Asian, and almost 5,000 Native American men.

We estimate the relationships first using Ordinary Least Squares (OLS) to show correlations among young men who served and did not serve in the Army and Army Air Force during World War II. The OLS method reduced potential selection bias by controlling for a variety of characteristics measured in 1940 before the war that likely influenced entry into the Army. To further control for selection bias for unmeasured factors, we take advantage of the age discontinuity related to eligibility created by the end of the war. Men inducted just before the war ended are the closest possible comparison group to men who did not become eligible for service until just after the war ended. This institutional feature has been exploited to create quasi-random variation in veteran status in previous work that used fuzzy regression discontinuity (FRD) designs (see Turner and Bound, 2003; Fetter, 2013). After controlling for the 1940 characteristics, the identifying assumption is that individuals with differential earnings potential could not affect or delay their induction around the end of the war, and that the cohorts just before and after the end of the war were not systematically different in terms of their future earnings potential.

In comparisons of Army World War II veterans with non-Army men, the OLS estimates with 1940 controls suggest that WWII Army veterans had higher incomes than non-Army men within the same group, and Army veterans were less likely to change counties between 1940 and 1969 than non-Army men within the Non-Hispanic White, Black, and Hispanic groups. To control for selection bias, we then estimate the effects with a fuzzy regression discontinuity design with the 1940 controls to compare men who were just too young to serve during World War II to men who were just old enough to become Army veterans. Those results showed that Army veterans had lower adjusted gross incomes than non-Army men within the Non-Hispanic White, Black, and Asian groups, and Army veterans had slightly higher incomes within the Hispanic and Native American groups. The differences varied by type of income. Migration across county boundaries was lower for Army veterans than non-Army men among Non-Hispanic Whites, Asians, and Native Americans and there were small differences for Black and Hispanic Army veterans compared to non-Army men.

In examining the impact of World War II Army service, our non-Army group includes men who served in World War II in the Navy, Marines, and Coast Guard and men who served in the Korean War. In the last section of the paper, we use an alternative data source to provide some evidence about what the comparisons of Army and non-Army men during World War II suggest for comparisons of all men who served in World War II to men who did not serve, while also accounting for service in the Korean War. We find that the OLS Army results understate the positive correlations for all men who served during World War II and the FRD Army results tend to be more negative than the ones for all men who served in World War II.

Our paper contributes to a growing literature on the effects of World War II on the U.S. labor market. Previous work has studied the effect of the war economy on the employment of women (Goldin, 1991; Acemoglu et al. 2004; Fernandez et al., 2004; Bailey and Collins, 2006; Goldin and Olivetti, 2013; Shatnawi and Fishback, 2018), and Black and White workers (Turner and Bound, 2003; Collins, 2006; Aizer et al., 2020; Ferrara, 2022).^2^ Our contributions join new research on differences for Blacks and Whites in this special issue of the journal by Collins and Zimran (2025) for outcomes in the 1950 Census and Lleras-Muney et al. (2025) for longevity. We also contribute to a literature seeking to better understand the impact of service on veterans’ labor market outcomes (Angrist and Krueger, 1994). Our paper provides new evidence for the impact of WWII service on Army veterans’ investment income, business and farm income, and total income, migration patterns, and the characteristics of the census tracts where they resided. More importantly, we highlight differences in the Army effect not only for Black and Non-Hispanic White men, but we also have sufficiently many observations to say more about the impact of WWII service on Hispanic, Asian, and Native American Army veterans, which have not received equal attention in previous work.

## 2. Historical background and related literature

More than 16 million Americans served during World War II, almost 10 million of whom were inducted via the draft. Even though America’s soldiers were predominantly male and Non-Hispanic White, other minority groups shouldered a significant part of the war burden. Around 350,000 women served during World War II. Over one million Black Americans, more than 13,000 Chinese Americans, 20,000 Japanese Americans, almost 20,000 Native Americans, and half a million Hispanic Americans served in the U.S. armed forces (Hershey, 1948; U.S. Congress, 2007).^3^

### 2.1. The World War II experience of Black Americans

The Selective Service System, which organized the mobilization of Americans for the war effort, was mainly designed and run by military planners with the help of local draft boards. A segregated military created issues for a successful draft, which most visibly affected Black Americans, the largest minority group in the U.S. at the time. Of the 6,442 draft boards with their approximate 25,000 board members,^4^ only 250 members across 33 states were Black, and in the South only Virginia, Kentucky, and North Carolina had any Black board members (Flynn, 1984).

This lack of representation was reflected in low early draft and enlistment rates among Black Americans. Prior to Pearl Harbor, there were only 4,450 Black soldiers in six regiments. The Marines did not accept Black volunteers until 1943, and the Navy restricted Black opportunities to messmen service. As late as January 1943, the share of Black soldiers in the Army was 5.9 % and in July of 1944, only 5 % of Navy sailors were Black (Flynn, 1984). The share of Black men drafted did not reach their 10 % share of the entire population until near the end of the war after several presidential Executive Orders had been issued to force all service branches to accept Black soldiers. Medical and education requirements were the most frequently cited reason for rejection of Black draftees.^5^ The Army’s inability to construct housing units to maintain segregation also contributed to higher rejection rates. An unintended consequence of the higher rejection rates – as well as the more frequent placement of Black men in support units rather than fighting units – was that Black soldiers experienced lower casualty rates (Ferrara, 2022).

Once employed in the few segregated fighting units, Black soldiers achieved remarkable successes on the battlefield.^6^ Not everyone was keen to join the military, however. Qian and Tabellini (2021) provide evidence that Black Southerners were less willing to volunteer when they experienced more discrimination in their home counties. Black community leaders, such as W.E.B. Du Bois, promoted volunteering because they argued that active participation in the war would strengthen their bargaining position when re-negotiating the social contract between Blacks and Non-Hispanic Whites in the postwar era (Parker, 2009). This was reflected in the Double-V campaign, which combined the victory over Nazism abroad and the victory over racism at home. Many Black servicemen experienced friendly treatment by their mostly White hosts in France and Britain (Schindler and Westcott, 2021). Yet, upon returning to the United States, they faced many of the same racial barriers from before the war. As a result, many Black veterans participated in the subsequent rise of the Civil Rights movement (Phillips, 2012).

Earlier studies have examined the impact of World War II service on the post-war experience of Black workers.^7^ There was substantial occupational upgrading of Black workers from mostly agricultural to industrial jobs during and immediately after the war due to labor shortages in various industries (Collins, 2000; Aizer et al., 2020; Ferrara, 2022). The upgrading contributed to the narrowing in the Black-White wage gap (Margo, 1995). Labor market gains were realized by both Black men and women (Bailey and Collins, 2006). In contrast, Collins (2000) does not find significant advantages for Black veterans in the labor market in terms of occupational upgrading or wages in a sample of six cities.^8^

The impact of the second Great Migration to the industrial centers also contributed to Black economic progress in the 1940s (Boustan, 2016; Derenoncourt, 2022). Even though it is not always linked to World War II explicitly, the need for additional labor after the war began with the rise of the war economy (Wolfbein, 1947). Black veterans could take advantage of the G.I. Bill and attend college, but these benefits mainly accrued to those living outside the South (Turner and Bound, 2003).

In the Collins and Zimran (2025) study the veteran effect on years of schooling in 1950 was stronger for Blacks than for Native Whites for men with less than 12 years of schooling in 1940. The veteran effect on employment in 1950 was more negative for Blacks than for Native Whites, in part because the veteran effect on school attendance in 1950 was larger for Blacks than for Native Whites. Among men reporting an occupation in 1950, the veteran effect on occupation scores was less positive for young Blacks than young Native Whites, but slightly more positive for older Blacks than for older Native Whites. The veteran effect on earned income was slightly less positive for young Blacks than for young Native Whites and was slightly negative and about the same for older Blacks and older Native Whites.

### 2.2. Asian, Hispanic, and Native Americans in World War II

Even though Black Americans were the largest of the minority groups, Hispanic, Asians, and Native Americans also made significant contributions to the war effort. Around 20,000 Native American soldiers served in the war and had the highest participation rate of any group (Holm, 1981). Their culture and language also contributed directly to the American war success, and the 29 original *Navajo Code Talkers*, who helped the Marines secure their lines of communications in the Pacific theatre, were awarded the Congressional Gold Medal on July 26, 2001 (Jevec, 2001).

The second largest minority group were Hispanics. Roughly 400,000 to 500,000 served during World War II. This range is an estimate because Hispanics were not listed as a separate racial group but treated as White soldiers in most studies and data sets (Converse et al., 2008). Their listing as Whites in the Census does not imply that they experienced no discrimination. A significant number of Mexican Americans, the largest subgroup among Hispanics at the time, had been deported during the Great Depression (Lee et al., 2019), only to be invited to return to work on American farms as labor shortages in the agricultural sector during the war threatened food supply.

Alongside German and Italian immigrants in the U.S., Asian Americans were the target of hatred, especially after the attack on Pearl Harbor. Japanese citizens and Japanese Americans were interned in camps, while discriminatory behavior spilled over to other Asian groups due to Americans’ inability to distinguish between different Asian groups (see Yui, 1992; Saavedra, 2021; Arellano-Bover, 2022).

There is relatively little systematic evidence for the labor market effects of the war and WWII service on the incomes and migration patterns of Hispanics, Asians, and Native Americans. Using the one-percent sample of the 1960 Census, Browning et al. (1973) conclude that Black and Mexican American veterans earned higher wages than non-veterans, which they attribute to the networks and knowledge gained from the military providing a bridge to better occupations. Their findings are called into question in terms of causality by Collins (2000) and a literature that attributes wage gains of veterans to selection bias into the military. The positive wage effects disappear once the analysis controls for selection bias (Angrist and Krueger, 1994).^9^ However, research on the impact of military service in the 2000s, suggests that the impacts of military service could differ by race. In a study of the Post 9/11 era, Greenberg et al. (2022) find that military service increased annual incomes 11 to 19 years later by $5,500 to $15,000 for Black service members, while White service members experienced no significant changes.

## 3. Data

We employ two methods to control for potential selection bias into the military: controlling for 1940 characteristics that influenced entry into military service, as done by Collins and Zimran (2025), followed by a fuzzy regression discontinuity (FRD) approach with those controls in which the age of military eligibility and the time frame of the war play an important role. The goal of the FRD is to compare men who were just old enough to serve to similar men who were just too young to serve. To operationalize the FRD, our sample is restricted to men born between 1923 and 1932. Men born in the year 1928 or earlier were eligible for World War II service until the age of 44, while those born later than 1929 were not eligible. The pre-treatment characteristics for Army veterans and non-Army men come from the full count 1940 Decennial Census made available by the U.S. Census Bureau and digitized by IPUMS (Ruggles et al., 2024a). Army veteran status is obtained by merging the full count census to records from the Army and Army Air Force using the World War II Enlistment Records. Information on outcomes later in life are obtained by merging these data with administrative records from the 1969 Internal Revenue Service (IRS) 1040 tax return form. We use the Census Bureau’s internal “best race” file to assign race and ethnicity to individuals (Ennis et al. 2018). The best race file uses an algorithm to compile race and ethnicity data from the decennial census, household surveys, and administrative records and then assigns race and ethnicity to everyone in a master file based on the quality of the race and ethnicity data and the proximity to the moment of observation.

The 1040 form contains data on individual wage and salary income, interest and dividend payments, adjusted gross income, and location of residence in 1969. We compare the respondent’s county and state of residence in the 1940 Census to their location in the 1969 IRS data to construct mobility variables. To see how the individual fits into their census tract of residence in 1969, we calculate the median wage and salary income and the percentage of residents of the same racial group in the Census tract where they resided.^10^ In addition, at the county level we construct a dissimilarity index in 1969 for Non-Hispanic Whites versus a group made up of all the minority groups.^11^ It measures the minimum percentage of people from the other group that would have to move to have the other group spread across the tracts in the county in the same way as the Non-Hispanic Whites were spread.

To link the 1940 Census to the IRS file, and military records we used the assignment of protected identification keys (PIKs) to each individual by the Census Bureau. These are administrative individual identifiers based on individuals’ social security numbers and other individual characteristics like name and date of birth that were introduced in 1936.^12^ The match rates between the 1940 complete census, the military records, and the IRS file were roughly 50–60 % or higher for the age group we study.

Table 1 presents summary statistics comparing the mean values of correlates before the war from the 1940 Census for male WWII Army veterans and non-Army men in the sample. The comparisons are dominated by comparisons for Non-Hispanic Whites, who account for roughly 90 % of the sample. Since the sample was designed for a fuzzy regression discontinuity framework, the average age of non-Army men was lower. All the men were 18 or younger by 1940; therefore, marriage rates were very low, 0.24 % for Army veterans and 0.11 for non-Army men. Army veterans averaged 2.7 years more of schooling and were 6.4 % more likely to be in the labor force.

There are smaller differences for less age-related correlates from 1940. The difference in income per household member was only $11.60. Army veterans were only 2.0 percentage points more likely to live in urban areas and 2.8 percentage points more likely to be residing in the same county as in 1935. They were 0.2 percentage points less likely to be US citizens and 2.2 percentage points more likely to be White than non-Army men.^13^ Additionally, Army veterans were more likely to come from the Northeast.

Table 2 presents summary statistics comparing the mean values of outcome variables from the 1969 1040 tax data for male WWII Army veterans and non-Army men. The Army veterans were 3.5 % less likely to be living in a 1969 county that differed from their 1940 county. They had higher incomes of all types; adjusted gross income was about $800 higher than for non-Army men. Army veterans and non-Army men lived in census tracts with similar median wage and salary incomes. The Army veterans and non-Army men both tended to live in census tracts where roughly 70 % of the tract population was of the same race, and both tended to live in counties with a dissimilarity index of around 0.23.

## 4. Methods

We seek to identify the causal relationship between participation in the military in World War II and outcomes later in life for each group. A simple comparison of Army veterans to non-Army men is likely to lead to problems with endogeneity and/or selection bias because a significant number of Army veterans volunteered, and the draft was not fully random. Further, the military put both volunteers and draftees through a selection process that weeded out men who could not perform military tasks. Collins and Zimran (2025) provide evidence that younger veterans “were positively selected on the basis of education and neutrally or negatively selected on the basis of their own or their father’s labor market characteristics.”

To address the problem of selection bias, we follow two strategies. The first follows Collins and Zimran (2025) by estimating an OLS regression on our sample while controlling for a number of characteristics from 1940 that influenced joining the military and the army. In that case we estimate a version of the Second-Stage equation (1) below in which we use the actual service in the Army rather than the predicted service.

Given that there are likely to be unmeasured characteristics that influenced Army service, we follow strategies developed by Turner and Bound (2003) and Fetter (2013) by using a fuzzy regression discontinuity (FRD) approach. This involves several steps. To reduce the endogeneity and selection bias, we limit the sample to men born between 1923 and 1932 and split them into two groups: those born from 1923 to 1928 and those born from 1929 through 1932. The group born in 1928 or before was eligible to participate in the military at some point during the war because they would have turned 17 before 1945. Age 17 was the cutoff for eligibility for the draft and volunteering during the war. Males born after 1928 would not have been eligible, although some might have joined by lying about their age.

We then create an instrument for WWII Army service, which is a zero-one dummy that has a value of one when the person was born in 1928 or before and therefore was eligible for the draft at some time during the war. It was zero for people born after 1928 who were thus not eligible at any time during the war. Essentially, the dummy is a measure of an intent-to-treat based on the age cutoff. To control for other differences that arise based on age, like job experience, we include a linear age trend variable that serves as a running variable across the cutoff birth year. We allow for this trend to differ on both sides of the cutoff year.

The FRD design is implemented by estimating a Two Stage Least Squares (2SLS) model on a pooled sample that contains members of all five groups. There are five first-stage equations that incorporate five instruments for serving in World War II that are based on the race-specific birth-year cutoffs, as well as one second stage equation. The five instruments include the birth year cutoff for all Army veterans and then four instruments that multiply the birth year cutoff for all Army veterans multiplied by a dummy variable for each of the four minority groups.

To simplify the notation and the description, consider a situation where the data set only has two groups, Non-Hispanic White and Black men. In that case, we would estimate a second-stage equation and two first-stage equations.

Second-Stage Equation 1) $Y_{ivsg}=\beta_{s}+\beta_{X}X40_{ivsg}+\beta_{v}W\hat{W2_{ivsg}}+\beta_{b}B_{ivsg}+\beta_{vb}BW\hat{W2_{ivsg}}\;+\beta_{r1}RUN_{ivsg}+\beta_{r2}RUN_{ivsg}\times B28_{ivsg}\;+\beta_{br1}RUN_{ivsg}\times B_{ivsg}+\beta_{br2}RUN_{ivsg}\times B28_{ivsg}\times B_{ivsg}+\varepsilon_{ivsg}$

In the second stage equation $Y_{isvg}$ is an outcome for individual i of racial or ethnic group *g* with World War II Army veteran status *v* and living in state *s* in 1940. We estimate the model for each outcome listed in Table 2 while taking the natural log of the income measures. There is a dummy variable $B_{ivsg}$ for Blacks. The reference category is Non-Hispanic White men. The coefficient for the Black dummy, $\beta_{b}$, is an estimate of the difference in the outcome between Black non-Army men and the outcome for Non-Hispanic White non-Army men.

State resident fixed effects in vector $\beta_{s}$ are included to control for 1940 state of residence. The control vector $X40_{ivsg}$ contains variables controlling for pre-treatment characteristics from the 1940 Census that were listed in Table 1, including per capita household wage and salary income, school enrollment status, years of schooling, employment status, labor force status, having moved between 1935 and 1940, urban status, marital status, and citizenship. We do not include Army veteran or Black interactions with the correlates, so we can use the coefficient on the Black dummy as a measure of the difference between outcomes for Black non-Army men and Non-Hispanic White Army veterans.

The predictions of WWII service in the second stage equation are $W\hat{W2_{ivsg}}$ for all Army veterans and $BW\hat{W2_{ivsg}}$ for Black Army veterans. The coefficient $\beta_{v}$ shows the difference in outcomes between Non-Hispanic White Army veterans and Non-Hispanic White non-Army men. The difference in outcomes between Black Army veterans and Black non-Army men is shown by the sum of the coefficients on the prediction of World War II service for all Army veterans and the Black prediction of World War II service, $\left(\beta_{v}+\beta_{vb}\right)$. Finally, the coefficient $\beta_{vb}$ on the prediction for Black Army veteran status shows a difference-in-difference calculation that is the difference between the impact of service for Blacks and the impact of service for Non-Hispanic Whites.

As part of the 2SLS procedure to control for endogeneity and selection, the actual World War II Army veteran status is replaced by the predicted probability of Army veteran status from two first-stage equations with two instrumental variables. The dependent variables are a dummy for World War II service for all men, $WW2_{ivsg}$, and a dummy for Blacks who served in World War II, $BWW2_{ivsg}$, which is the equivalent of multiplying $WW2_{ivsg}$ by a Black dummy variable.

First-Stage equations.2a) $WW2_{ivsg}=\delta_{s}+\delta_{X}X_{ivsg}+\delta_{v}B28_{ivsg}+\delta_{b}B_{ivsg}+\delta_{bv}B28_{ivsg}\times B_{ivsg}\;+\delta_{wr}RUN_{ivsg}+\delta_{wr}RUN_{ivsg}\times B28_{ivsg}\;+\delta_{br}RUN_{ivsg}\times B_{ivsg}+\delta_{br}RUN_{ivsg}\times B28_{ivsg}\times B_{ivsg}+u1_{ivsg}$

2b) $$BWW2_{ivsg}=\delta_{sb}+\delta_{Xb}X_{ivsg}+\delta_{vb}B28_{ivsg}+\delta_{bb}B_{ivsg}+\delta_{bvb}B28_{ivsg}\times B_{ivsg}+\;+\delta_{br}RUN_{ivsg}+\delta_{br}RUN_{ivsg}\times B28_{ivsg}\;+\delta_{bbr}RUN_{ivsg}\times B_{ivsg}+\delta_{bbr}RUN_{ivsg}\times B28_{ivsg}\times B_{ivsg}+u2_{ivsg}$$

As another feature of the fuzzy regression discontinuity design, each of the instruments in the first-stage equations is a dummy with a value of one for men who were born before or during 1928 and zero from 1929 onward. The dummy for all Army veterans is $B28_{ivsg}$ and for Blacks it is the interaction between the dummy for all Army veterans and the Black dummy, $B28_{ivsg}\times B_{ivsg}$. Another part of the design is the inclusion in the second-stage equation of a running variable, $RUN_{ivsg}$, that subtracts the cutoff year 1928 from the birth year for the observation. We allow for different trends before and after the cutoff year with an interaction term, $RUN_{ivsg}\times B28_{ivsg}$, which is the product of the running variable and the dummy for men born in 1928 or earlier. We also allow for different trends before and after the cutoff for Blacks with the interaction terms, $RUN_{ivsg}\times B_{ivsg}$ and $RUN_{ivsg}\times B28_{ivsg}\times B_{ivsg}$. Although we illustrated the procedure here with just two groups, we run the model with 5 groups by adding Hispanics, Asians, and Native Americans to the data set and adding dummies and interaction terms for each of the three additional groups.

Fig. 1 shows the relationship between birth year and Army service in World War II for two groups: non-Hispanics Whites and the grouping of Blacks, Hispanics, Asians, and Native Americans. For both groups the probability of serving in the Army during the war declines as the birth year rises toward 1928. For men born five years before 1928 the share of non-Hispanic White males serving in the Army was approximately 34 % and the share of non-White and Hispanic males serving in the Army was around 27 %. These rates fell to around 11 to 12 % for men born in 1928. The shares for both when born in 1929 and thus ineligible for service were less than three percent and declined to near zero for later birth years.

There may be some measurement error in the ages reported in the 1940 Census, particularly for minorities, because people were less likely to have their birth certificates before 1950 (Eriksson et al., 2018).^14^ The errors could lead to age heaping in which the number of people with ages divisible by five are over-stated. Note that the age heaping is not a problem for our estimation if there is no difference in the age heaping between the men who later became World War II Army veterans and non-Army men.

To check for possible age heaping, Fig. 2 shows the distributions of ages for each group in the sample. In the 1940 Census people reported the person’s age at their last birthday at the end of March. People reporting age 15 in 1940 would have been referring to roughly three-fourths of the boys born in 1924 and one-fourth of the boys born in 1925; those reporting age 10 would have referred to three-fourths of the boys born in 1929 and one-fourth of the boys born in 1930. Thus, age heaping would have had the largest positive effect relative to other years on the number of boys reported with birth years 1924 and 1929 and a smaller relative positive effect on the number with birth years 1925 and 1930. Our cutoff focused on males born in 1928 or before. In the distributions in Fig. 2, the 1929 and 1930 shares of observations are not unusually large relative to 1928 for four of five groups. The exception was the distribution for Native Americans, which is unusual among the distributions because it shows such a large rise in the number born after 1926. For all groups, the share born in 1924 does tend to be larger than the share in 1923, but this is not near the cutoff and likely has little effect on the analysis.

Age measurement issues might also create problems for the 1928 cutoff. To examine the robustness of the 1928 cutoff, we performed the structural break estimation technique used in Chay et al. (2005), Card et al. (2008), and Fetter (2013). This technique required us to randomly select 30 % of the sample, to test how various cutoffs perform in terms of model fit, while using the remaining 70 % of the sample for estimation. We used the technique for the pooled sample with all racial and ethnic groups included and then for subsamples based on race, ethnicity, and region. Overall, the method suggested that the 1928 cutoff was preferred to other year cutoffs and a race by region specific cutoff based on a higher R-squared than the other contenders.

Fig. 3 shows examples of how one of our outcomes, a change in county between 1940 and 1969, was related to the birth year without controlling for other factors. We use it to illustrate the difference between the FRD analysis and an OLS analysis without any other controls. The vertical line at birth year 1928 shows the intent-to-treat cutoff where only males born in 1928 or earlier were eligible for World War II service. The OLS estimation would focus on the difference in means between the 1923–1928 period for men eligible for service and the 1929–1932 period for those individuals who were ineligible. In an analysis with controls, the OLS would have also controlled for age. The FRD design puts the highest weight on the change between birth year 1928 and 1929 and lower weights as the birth years are farther apart. The difference in trends related to year of birth before and after the cutoff for four of the 5 groups are the reasons why we included separate trends in the running variables in the analysis.

For Non-Hispanic White men, the difference in means suggests that the men eligible for service had a probability of moving that was about three percentage points below the probability for the men who were ineligible, while the FRD analysis puts the most weight on the 1928–1929 difference in which the men eligible for service were one percentage point less likely to move. For Blacks, the means for eligible and ineligible men were very similar, while the FRD puts the most emphasis at the cutoff where the eligible men were roughly 0.5 percentage points more likely to move. Among Hispanics, the difference in means suggests that the eligible men were three percentage points less likely to move, while the FRD emphasizes the lack of difference at the cutoff. Among Asians the means were about the same, while the FRD puts the most emphasis on the cutoff where the eligible men were about two percent less likely to move. For Native Americans the difference in means implied that the eligible men were about 0.7 percentage points less likely to move. The change at the cutoff suggests that they were about 2 percentage points less likely to move.

## 5. OLS estimates

The OLS estimation works to control for selection bias by controlling for individual and family characteristics from 1940 just before the war. These results provide rough comparisons of our findings for 1969 outcomes with the findings for 1950 outcomes in OLS estimations by Collins and Zimran (2025) for young men in this issue. The comparisons are rough for two reasons. First, Collins and Zimran (2025) compare men born from 1921 through 1927 who served to the men born between 1921 through 1927 who did not serve. Since our OLS estimation is designed to match the sample in the FRD analysis, the comparison is between men who served in the Army who were born from 1923 through 1928 to men who were born in that period who did not serve in the Army and men who *could* not serve because they were born from 1929 through 1932. Second, the studies use different but overlapping lists of correlates.^15^

### 5.1. Comparisons of the minority non-Army outcomes with non-Army outcomes of Non-Hispanic Whites

Table 3 provides estimates of the differences in outcomes between minority non-Army men and Non-Hispanic White non-Army men while controlling for the correlates from the 1940 Census listed in the notes to the table. The results here give a sense of the situation for those who had not served in the Army, although their situations would have been indirectly affected by the activities of the Army veterans. As in many other situations, the results show that the incomes of the minority groups were lower than for Non-Hispanic Whites with some exceptions. Asians were more likely than Non-Hispanic Whites to report interest and dividend and business or farm income. Among nonveterans reporting positive amounts of income, Asians had higher business/farm income, and their overall incomes were only slightly lower.

Black non-Army men tended to move more than Non-Hispanic White non-Army men, particularly longer distances. They were 4 percentage points more likely to live in a different county in 1969 than in 1940, and they were more than 15 percentage points more likely to switch states, census districts and census regions. Asian non-Army men were more than 4.6 percentage points more likely than Non-Hispanic White non-Army men to move across states, districts, and regions, but only 1.5 percentage points more likely to change counties. Hispanic non-Army men were about 6 percentage points less likely to change counties than Non-Hispanic White men but were more than 1.6 percentage points more likely to change states, districts, and regions, while Native American non-Army men and Non-Hispanic White non-Army men displayed similar patterns of movement.

Among non-Army men all four minority groups lived in census tracts in 1969 with substantially lower median incomes than for Non-Hispanic White men. Blacks, Hispanics, and Asians all lived in counties where the dissimilarity index was higher than for Non-Hispanic White men. The dissimilarity indices were similar on average for Native Americans and Non-Hispanic White men.

### 5.2. OLS comparisons of veteran and non-veteran outcomes within the same racial groups

The OLS estimates in Table 4 of the differences in incomes between Army veterans and non-Army men within the same group are generally positive. We describe the point estimates. The ones that are statistically significant at the 10-percent level are marked with an asterisk. Within each of the 5 groups Army veterans were more likely than non-Army men to file a tax return in 1969 and to report positive interest/dividend; the gap is positive on reporting business/farm incomes for all but the Black group. Within each group of men having reported a positive amount of income in each category, Army veterans tended to have higher wage and salary incomes and adjusted gross incomes than non-Army men.^16^ Collins and Zimran (2025) found that young veterans in 1950 earned higher wages and salaries than non-veteran men although the Black coefficient was not statistically significant. For older men the veteran effect was slightly negative and statistically insignificant. For the other components of income, Black, Hispanic, and Asian veterans had higher business/farm incomes than their non-veteran counterparts. Native Americans and Non-Hispanic White veterans tended to have higher interest/dividend income than their non-veteran counterparts.

Army service caused nearly all the men who served to travel to other places during their time in service, which might have led Army veterans to make more long-term decisions to move than non-Army men. However, the results show that Army veterans were less likely than non-Army men to have changed counties within each of the Non-Hispanic White, Black, and Hispanic groups. Among Asians, Army veterans were 2.4 percentage points more likely to move across states than non-Army men, while Army veteran Native Americans were more than 4.1 % points more likely to change states, districts, or regions than non-Army Native Americans.

In the locations where they lived, all but the Non-Hispanic White Army veterans lived in census tracts with higher median wages than the tracts where their non-Army counterparts lived. Finally, for each group there was little difference between Army veterans and non-Army men in the percentage of the same group living in the census tract or in the county dissimilarity index where Army veterans and non-Army men of the same group lived.

## 6. Fuzzy regression discontinuity results

The FRD is designed to reduce problems with selection into the Army while controlling for 1940 characteristics when examining outcomes later in life. Table 5 presents 2SLS estimates of the five first stage equations. The coefficient in column (1) of 0.104 for all men born in 1928 or before in the first line implies that the probability of service among all men was 10.4 percentage points higher than for men born after 1928. There are also statistically significant coefficients for the Asian and Black interaction terms. The F-statistic for the five instruments as a group is 6,830, which is well above thresholds for instrument strength. In column (2), the coefficient of 0.066 for the Black interaction with the birth year variable is statistically significant. The other coefficients are all much smaller in magnitude and all but one are statistically insignificant. The situations for the other three groups are like the Black situation because they also have strong positive coefficients for the group-specific interaction and small effects for the other instruments. The only equation where instrument strength might be an issue is for Native Americans because the F-statistic for the five instruments is 8.18.

These results are estimated for the full sample of tax return filers. When we estimate models for specific outcomes with fewer observations, the first stage uses only those observations. The results in those first-stage estimations are similar to the results in Table 5.

Tables 6 and 7 summarize the second-stage results for the FRD analyses of equation 1 for 35 outcomes. The coefficients and standard errors underlying these Tables are reported in Appendix Tables 1–1 through 1–7. The estimates in the row for an outcome in Tables 6 and 7 come from the same estimation procedure. In row 1 of Table 6, the coefficients compare the difference in outcomes between Army veterans and non-Army men of the same group. The −0.079 coefficient for the Non-Hispanic White men is the $\beta_{v}$ coefficient in equation 1 and shows that Non-Hispanic White Army veterans were 7.9 percentage points less likely to file 1969 Tax Returns than Non-Hispanic White non-Army men. The coefficient of –0.081 in the Black column is the sum of the coefficients $\beta_{v}$ and $\beta_{vb}$ in equation 1 and shows that Black Army veterans were 8.1 percentage points less likely to file a return than Black non-Army men. The coefficients for the other minority groups also show that Army veterans were less likely to file than non-Army men.

The coefficient in the Black column of Row 1 in Table 7 shows the coefficient equivalent to $\beta_{vb}$ in equation 1, which is a difference-in-difference calculation that shows the difference between the Army/non-Army difference for Blacks and the Army/non-Army difference for Non-Hispanic White men. The Black coefficient of −0.002 in the first row shows virtually no difference between the Army effects for filing tax returns for Blacks and Non-Hispanic Whites. The Hispanic and Asian gaps are more positive than for Non-Hispanic Whites, while the Native-American gap is more negative, although none of the coefficients are statistically significant.

### 6.1. Differences in outcomes for Army veterans versus non-Army men for non-Hispanic White men

Non-Hispanic White men were the group least likely to experience discrimination; therefore, we later use their outcomes as the baseline for comparisons of the Army/non-Army gaps or the minority groups. The differences between the Army and non-Army experiences of the Non-Hispanic White men are summarized in the column labeled “Non-Hispanic White” in Table 6.^17^ Most of the coefficients are statistically significant in two-tailed tests at the 90-percent level or above.

The FRD results for income contrast sharply with the positive Army veterans’ findings in the OLS estimations in Table 4 and the findings for young men in 1950 in Collins and Zimran (2025). Among Non-Hispanic Whites, Army veterans fared poorly in comparisons to non-Army men. Army veterans were 7.9 percentage points less likely than non-Army men to file a 1969 tax return. Army veterans’ adjusted gross incomes were 0.079 log points lower, partly because their wage and salary incomes were 0.038 log points lower. The tax return dataset provides the first opportunity to see the investment and business/farm activities of Army veterans and non-Army men. Army veterans were also 7.0 percentage points less likely to report interest and dividend income and 1.6 % less likely to report farm or business incomes. When they did report those incomes, their dividend/interest income and business/farm incomes were 0.19 and 0.34 log points lower than for non-Army men.

Although serving in the war forced veterans to travel, the FRD results show that Army veterans were less likely than non-Army men to reside in a different location in 1969 than before the war in 1940. These FRD results are more negative than the OLS results in Table 4. Army veterans were 5.1 percentage points less likely to have changed counties, and they were more than 10 percentage points less likely to have changed states, census district, or census region. The Army veterans lived in census tracts with median tract earnings were $385 lower than where non-Army men lived There was little difference in the share Non-Hispanic White population in the tracts where Army veterans and non-Army men lived, nor was there much difference in the dissimilarity index in the county where they resided.

Movers and stayers can often have different characteristics, so we split the dataset into movers and stayers and then compared the differences between Non-Hispanic White Army veterans and non-Army men among movers and then separately among stayers. The Army/non-Army difference in adjusted gross income among movers was less negative at −0.047 log points than it was for stayers at −0.113 log points. The smaller negative differences largely came about because the Army/non-Army gap for movers was substantially less negative lower for dividend/interest income and farm/business income. There were not large differences in the Army/non-Army gaps for movers and stayers in the characteristics of the locations where they lived.

### 6.2. Outcomes for Black Army veterans relative to Black non-Army men

In contrast with the OLS results that Black Army veterans had higher incomes than Black non-Army men, the FRD results in Table 6 under the Black heading show that the incomes of Black Army veterans were substantially lower than for Black non-Army men in every category. Among Blacks who reported positive amounts of each type of income, Army veterans reported substantially lower incomes than non-Army men; 0.33 log points lower for adjusted gross income, 0.18 log points lower for wage and salary income, 0.23 log points lower for interest/dividend income, and even worse for business/farm income. This is in line with the findings by Angrist and Krueger (1994) who reported a positive effect for the OLS but a negative one for the IV estimates, highlighting the potential of selectivity into service driving the OLS wage results.

The differences between Black Army veterans and Black non-Army men related to migration and the features of where they lived in 1969 were not statistically significant, although the magnitudes of some of the coefficients were large. Black Army veterans and Black non-Army men moved to a different county between 1940 and 1969 at virtually the same rate, but Army veterans were 2.6 % more likely to switch states and 7.7 % more likely to switch census regions. In the census tracts where they lived in 1969, Black Army veterans lived in tracts with $159 less in median income than Black non-Army men. Black Army veterans lived in slightly less segregated areas since the percent Black in the census tract where Black Army veterans lived was 3.4 % lower than in the Black non-Army tracts and the Black Army veterans lived in counties with a dissimilarity index that was 2.2 % lower.

Some differences arise when we split the sample into movers and stayers and compare Black Army veterans and Black non-Army men within each migration group. The Army/non-Army gaps for all types of income were less negative for movers than for stayers. For example, the Army/non-Army gap adjusted gross income was less negative for movers at −0.30 log points than for stayers at −0.42. The gaps were substantially less negative for movers than for stayers for dividend and interest income and for business and farm income. In the areas where they resided, the Army/non-Army gap in tract median incomes was more negative for movers at -$201 than for stayers at -$22. Among movers, Black Army veterans lived in tracts where the percent Black was 2.4 % lower than in tracts where the non-Army men lived, while the Black Army veterans lived in counties with 1.3 lower dissimilarity indexes. The Army/non-Army gaps among stayers were more negative at −6.1 % and −5.9, respectively.

The difference-in-difference coefficients in Table 7 show the difference between the Army/non-Army gap for Blacks and the Army/non-Army gap for Non-Hispanic Whites. When the coefficient is positive, World War II service helped reduce the overall gaps between Black and Non-Hispanic White men. The coefficients on the income measures for those who reported positive incomes were generally negative, as World War II Army service had even more negative effects for Blacks than the negative effects for Non-Hispanic White men in Table 5. Army service had a more positive effect on the movement of Blacks because the Non-Hispanic White veteran effect was negative while the Black veteran effect was positive.

### 6.3. Outcomes for Hispanic Army veterans relative to Hispanic non-Army men

Among Hispanics, Army veterans had slightly higher overall incomes than non-Army men in both the OLS results in Table 4 at 0.018 log points and in the FRD results in Table 6 at 0.008 log points. Most of the Army veterans’ slight advantage in the FRD results came from 0.044 log points higher wages and salaries, which offset their substantially lower interest/dividend incomes and farm/business incomes. The Army/non-Army differences in migration across state and regional boundaries in the FRD estimation are not statistically significant, but the magnitudes were more negative in the FRD estimations than in the statistically significant OLS results.

Median wage and salary incomes in the Census tracts where Army veterans resided were $188 lower than in non-Army tracts. The share Hispanic in the tracts where Army veterans lived was 2.4 percentage points lower than where non-Army men lived, but the dissimilarity index was roughly the same in the counties where both groups resided.

There are some dramatic differences in the Army/non-Army gaps when we compare results for movers and stayers among Hispanics. The Army effect on wage and salary income and adjusted gross incomes were positive 0.19 and 0.13 log points for stayers, compared with negative Army effects for movers of −0.106 and −0.114 log points. However, the Army effect on business and farm income was strongly positive for movers and strongly negative for stayers. The Army effect on tract median incomes where the Hispanics lived in 1969 was much more negative for stayers than for movers. The Army effect on the percentage of Hispanics living in the census tract was −4.9 % for movers but essentially zero for stayers.

The difference-in-difference coefficients in Table 7 show that the Hispanic Army effects for Hispanic wage and salary and adjusted gross incomes were 0.08 and 0.09 log points more positive than the effects on Non-Hispanic White men. The business/farm income Army/non-Army gaps for Hispanics were substantially less negative than for Non-Hispanic White men, while the interest/dividend income gaps for Hispanics were substantially more negative. The Army effects on migration were less negative for Hispanics than for Non-Hispanic White men. In the tracts where they resided, the Army/non-Army difference in the median census tract wage and salaries was less negative for Hispanics than for Non-Hispanic White men, while the differences in the county dissimilarity index were about the same for Hispanics and Non-Hispanic White men.

### 6.4. Outcomes for Asian Army veterans relative to Asian non-Army men

Among Asians the move from OLS to FRD switched the signs of the Army effects on the income measures and the migration measures from nearly all positive to nearly all negative, although only one of the FRD estimates are statistically significant. The Army effect for adjusted gross income shifted from 0.063 log points under the OLS to a statistically insignificant −0.057 log points. The Army effects for the components of income fell sharply with the switch from values ranging from −0.02 to 0.151 in the OLS to FRD values from −0.197 to −0. 332 log points. The migration measures shifted from Army veterans switching locations up to 2.4 % more than non-Army men under the OLS to the Army veteran gap ranging from −4.9 to 2.1 % in the FRD estimation.

Among Asians the Army/non-Army gaps for all the income measures were substantially more negative for the stayers than for the movers, even though the Army veteran gap in reporting positive interest/dividend income and business/farm income was more positive for the stayers. The Army effect on tract median incomes where the Asians lived in 1969 was much more negative for movers than for stayers. The Army effect on the percentage of Asians living in the census tract was −1.8 percentage points for movers and −3.1 percentage points for stayers.

The difference-in-difference coefficients in Table 7 show that the Army effects for Asian wage and salary incomes were 0.224 log points more negative than the effects for Non-Hispanic White men, but the Asian Army effect was 0.02 log points less negative than the effects for Non-Hispanic White males on adjusted gross income. The business/farm income and interest/dividend Army/non-Army gaps for Asians were within 1.1 percentage points of the negative gaps for Non-Hispanic White men. The Army effects on migration were substantially less negative for Asians than for Non-Hispanic White men. The Asian Army effects on the share Asian in the census tracts were 1.9 % less negative than the Non-Hispanic White Army veteran effect on the share Non-Hispanic White individuals in the census tract, and the Asian veteran effect on the dissimilarity index for the county was 0.044 lower than for Non-Hispanic White men.

### 6.5. Outcomes for Native American Army veterans relative to Native American non-Army men

Native Americans were the only group for which the Army/non-Army difference in adjusted gross income was more positive in the FRD estimation than in the OLS estimation. The respective differences of 0.041 log points and 0.019 log points, however, are not statistically significant in either case. In fact, all the Army effects on income under OLS and FRD are statistically insignificant except for the OLS estimate of 0.038 for filing a tax return. When the estimation moves from OLS to FRD the Army effect on wage and salary income turns negative from 0.031 to −0.054. Meanwhile, the business/farm income Army effect stays negative, and the interest/dividend effect stays positive but the magnitudes are substantially larger.

The FRD Army effect for migration had the same signs as in the OLS estimation but the FRD effect for changing counties was much more negative while the FRD effects for changing states and regions were much more positive. The FRD estimates show that Army veterans were 15.6 percentage points less likely to change counties between 1940 and 1969, but they were more than 15.9 % more likely to switch states and regions. The Army veterans relative to non-Army men lived in census tracts with dramatically lower median wages. The Army veterans also tended to live in counties where the dissimilarity index was substantially lower than the counties where the non-Army men lived.

There were several substantial differences in the Army veteran effects for Native American movers and stayers. The Army effects on wages and salaries and adjusted gross incomes were strongly positive for movers and strongly negative for stayers. The Army effects were more positive for stayers than for movers on reporting interest/dividend income and reporting business/farm income, and the Army effect on the amounts reported was more positive for stayers for interest/dividend income and less negative for movers for business/farm income. The Army effects for movers and stayers with respect to median earnings in the census tracts where they lived were similar. The Army effect on the share of Native Americans living in the tract was much more negative for stayers, while the Army effect on the county dissimilarity index was much more negative for stayers than for movers.

In the comparisons of the Army effects for Native Americans and Non-Hispanic White men, the Army effects on filing taxes and reporting non-wage income were more negative for Native Americans than for Non-Hispanic White men. Army veterans fared better in adjusted gross income and non-wage income among Native Americans than among Non-Hispanic White men. In migration comparisons the Army effect for Native Americans was more negative than for Non-Hispanic White men in switching counties, but much more positive when switching states and regions. In the areas where they lived, the Native American Army effect on census tract income averages was much more negative than the Non-Hispanic White Army effect, as were the Native American Army effects on the county dissimilarity index.

## 7. What does the Army service analysis predict for all who served in World War II?

We have focused on the impact of serving in the Army and Army Air Force because we had complete records on service in those two branches during World War II that were matched to the 1940 Census and the 1969 Tax Returns. We did not have records for those who served in the Navy, Marines, and Coast Guard during World War II. We also do not know which men in the sample served in the Korean War. As a result, we are comparing men who served in the Army and Army Air Force during World War II to three types of men: those who served in the Navy, Marines, and Coast Guard during World War II, those who served during the Korean War, and men who did not serve in either war.

Using information from the 5 % census sample for 1960 from the IPUMS, we can make some educated guesses about how the Army service results might be used to predict the impact of military service of all kinds during World War II (Ruggles et al., 2024a). We restricted the sample for 1960 in roughly the same way that we constructed the data set in the paper. We did not have access to names to match the 1960 information to our data set. Since non-Hispanic White males are the largest group in our analysis, we focused on that group in this IPUMS analysis. We eliminated people who lived in group quarters, men born before 1923, men born after 1932, and men who had no wage and salary income and those who were at the top-coded maximum.

Among the men in the 1960 IPUMS sample born from 1923 to 1928, 65.2 % served only during the World War II period between September 1940 through July 1947, 7.7 % served only during the Korean War period defined as June 1950 through January 1955, and 6.4 % served in both periods. Among the men in the IPUMS sample born from 1929 through 1932, 58.3 % served only during the Korean War period, 3.4 % served only during the World War II period, and around 1.1 % served in both periods.

Since 70 % of the men who served in the military during World War II were in the Army, we created a simulated army dummy variable by using a random number generator to treat 70 % of the men who served in the military in World War II as having been in the army.^18^ We then ran three OLS regressions that included birthplace location fixed effects and the running variables used in the 1969 regressions. The first regression included the Army dummy, the second included a dummy for military service during World War II, and the third included the World War II military service dummy and a dummy for service during the Korean War. The results of the OLS regressions are reported in the first three specifications in Table 8. One thing to note in all six specifications is that we are not controlling for a variety of factors from 1940 that we controlled for in the results reported in Tables 3 through 7.

In the OLS analysis the coefficient for the Army dummy, which is the measure we are using in the paper, understates the value for all military service men during World War II. When we only have the Army dummy in specification 1 the Army coefficient is 0.12. Since the non-Army men include men who served in the non-Army branches during World War II, we expect the Army coefficient to be lower than the coefficient for all World War II service men in specification 2. The results show that the Army coefficient in specification 1 is 59 % of the value of the coefficient of 0.202 for the World War II dummy in specification 2. Controlling for Korean War service in specification 3, increases the World War II coefficient to a small extent to 0.218, and the Army coefficient from specification 1 of 0.12 is 55 % of the WWII coefficient in specification 3.

In the IV analysis we used the FRD method for the Army variable in specification 4, and we used it for the military service variable during World War II variable in specifications 5 and 6. In specification 6 we used the Korean War dummy as a control and did not try to instrument for serving during the Korean War. As found in the comparison of OLS and IV coefficients in Tables 4 and 6, the IV coefficients are more negative than the OLS coefficients. Among the IV results in specifications 4 through 6, the coefficient of −0.04 when only the Army dummy is in the analysis in specification 4 is the most negative. When we can add back the nonarmy men to look at all who served in the military during World War II in specification 2, the coefficient is −0.028. When we then take the next step of controlling for Korean War service, the coefficient is 0.008 and not statistically significant.

In the final analysis the results are suggestive that the OLS Army coefficients we estimated in Tables 4 understate the positive correlation between serving in the military and wage and salary earnings, primarily because the non-Army men serving during World War II are part of the comparison group. Meanwhile, the IV coefficients using the Army dummy in Table 6 are more negative than the coefficients would be if the analysis focuses on all military service during World War II and controls for Korean War service. The main take-away of this exercise is that if we were to use the Army results as estimates for all WWII veterans, it would lead to an attenuation bias in the OLS estimates and an inflation bias in the IV estimates as predicted by econometric theory (see Bingley and Martinello, 2017; Ferrara et al., 2024). If we had information for all WWII veterans from other service branches are accounted for, the impact of the omitted Korean War veterans on results using either estimator is less damaging in terms of the potential for bias in either direction.

## 8. Conclusion

By combining the full census of 1940, military records, and the income tax records of 1969, we have been able to examine how serving in the Army and Army Air Force during World War II affected a variety of outcomes 24 years after the end of the war. The outcomes include incomes from wages and salaries, investments, businesses and farms, and the adjusted gross income; migration across counties, states, and regions; and descriptions of the locations where the people lived in 1969. Because the Army veteran experience likely differed for the most common group and for minorities, we compare outcomes for Army veterans and non-Army men within five groups: Non-Hispanic Whites, Blacks, Hispanics, Asians, and Native Americans. We also compare the Army/non-Army differences of each minority group to the same differences for the largest group, i.e. Non-Hispanic White men.

As a starting point, Ordinary Least Squares (OLS) reduced the extent of selection bias using controls from 1940 that likely influenced the men’s entry into the army. The Ordinary Least Squares results show generally positive effects of Army veteran status within each group. Army veterans within each group earned higher wages and salaries and higher adjusted gross incomes than their non-Army counterparts. The migration section of the OLS outcomes showed that Army veterans were between 0.6 % to 3.9 % less likely to live in different counties, states, and regions in 1940 and 1969 than their non-Army counterparts among Non-Hispanic Whites, Blacks, and Hispanics. Army veterans among Native Americans were over four percentage points more likely than non-Army men to move across state and regional boundaries, and the positive veteran effect for Asians was around two percentage points.

Since there may have been selection bias related to unmeasured factors that influenced whether someone entered the Army, we added an additional step to correct for selection bias using a Fuzzy Regression Discontinuity Design (FRD). The method uses the age cutoff for eligibility for service among young men as an instrument for actual service. Men born before 1929 were eligible for service at some point in the war, while men born later were not eligible.

Comparisons of the OLS and FRD results suggest that the process through which men joined the Army during World War II led to the selection of men into the Army with more skills and aptitude on dimensions that we are unable to measure. In contrast to the positive veteran effects in the OLS results, the FRD results show that Army veterans earned lower wages and salaries than non-Army men within the Non-Hispanic White, Black, Asian, and Native American groups; the most negative difference was −0.26 log points among Asians. The Army veteran’s adjusted gross incomes were lower than the non-Army value among Non-Hispanic Whites, Blacks, and Asians, as the largest negative gap was among Blacks at −0.33 log points.

There are several potential reasons why Army veterans would have been earning less than their non-veteran counterparts. One possibility is that the veterans faced substantial delays in schooling and peace-time job experience that would have slowed their advancement through the job hierarchy. A second is that the skills learned in combat and in combat support might not have translated well to peace-time production. A third is that many veterans experienced substantial psychological damage from experiencing the horrors of war that likely hindered their productivity. A fourth possibility is that physical disability from the war reduced their productivity. This is an omitted variable in the datasets that we have available to us.

The direction of the selection bias varied more for the migration variables. The FRD estimates of the Army effects for changing location were 3 to 10 percentage points more negative for Non-Hispanic White men than the OLS estimates, and the FRD veteran effects among Hispanics was roughly 3.0 percentage points more negative than the OLS veteran effects for moves across states. In contrast, among Blacks, the FRD results show that Army veterans were more likely to cross state and regional lines than non-Army men, while the OLS results suggested that Army veterans were generally less likely to move. The Army effects for Native Americans were substantially more positive for crossing state and regional boundaries in the FRD estimation than under the OLS. Meanwhile, the movement of Asian Army veterans relative to non-Army men was slightly more negative under the FRD estimation than under the OLS.

The FRD/OLS differences in results also extended to the characteristics of the places where the men lived in 1969. The OLS results implied that the Army veterans in each group tended to live in census tracts where median earnings were higher than or similar to the earnings in the census tracts where non-Army men lived. The FRD results are the complete reverse with Army veterans living in areas with substantially lower incomes than the areas where non-Army men lived. The OLS and FRD estimates of the Army effects for segregation patterns were similar for Non-Hispanic Whites, Blacks and Hispanics. Among Asians and Native Americans, the FRD results suggested that veterans tended to live in areas with a lower county dissimilarity index.

The analysis for the Army compares men who served in the Army and Army Air Force during World War II to men who did not serve in the military in World War II and Korea, men who served in the Marines, Navy, and Coast Guard in World War II, and men who served in Korea. A preliminary examination of an IPUMS census sample from 1960 with military service information suggests that our OLS results for the impact of Army service during World War II are less positive and the FRD estimates are more negative than the likely impact for all men who served during World War II. This issue can be resolved to a much greater extent when the World War II records for individual service outside the Army and Army Air Force become available, or the 1960 and 1970 full censuses, which asked questions about veteran service of 15–20 % of households, are fully digitized and numeric codes for individuals are attached by the Census Bureau.

## Supplementary Material

supplementary materials

Supplementary material associated with this article can be found, in the online version, at doi:10.1016/j.eeh.2025.101687.

## Figures and Tables

**Fig. 1.**
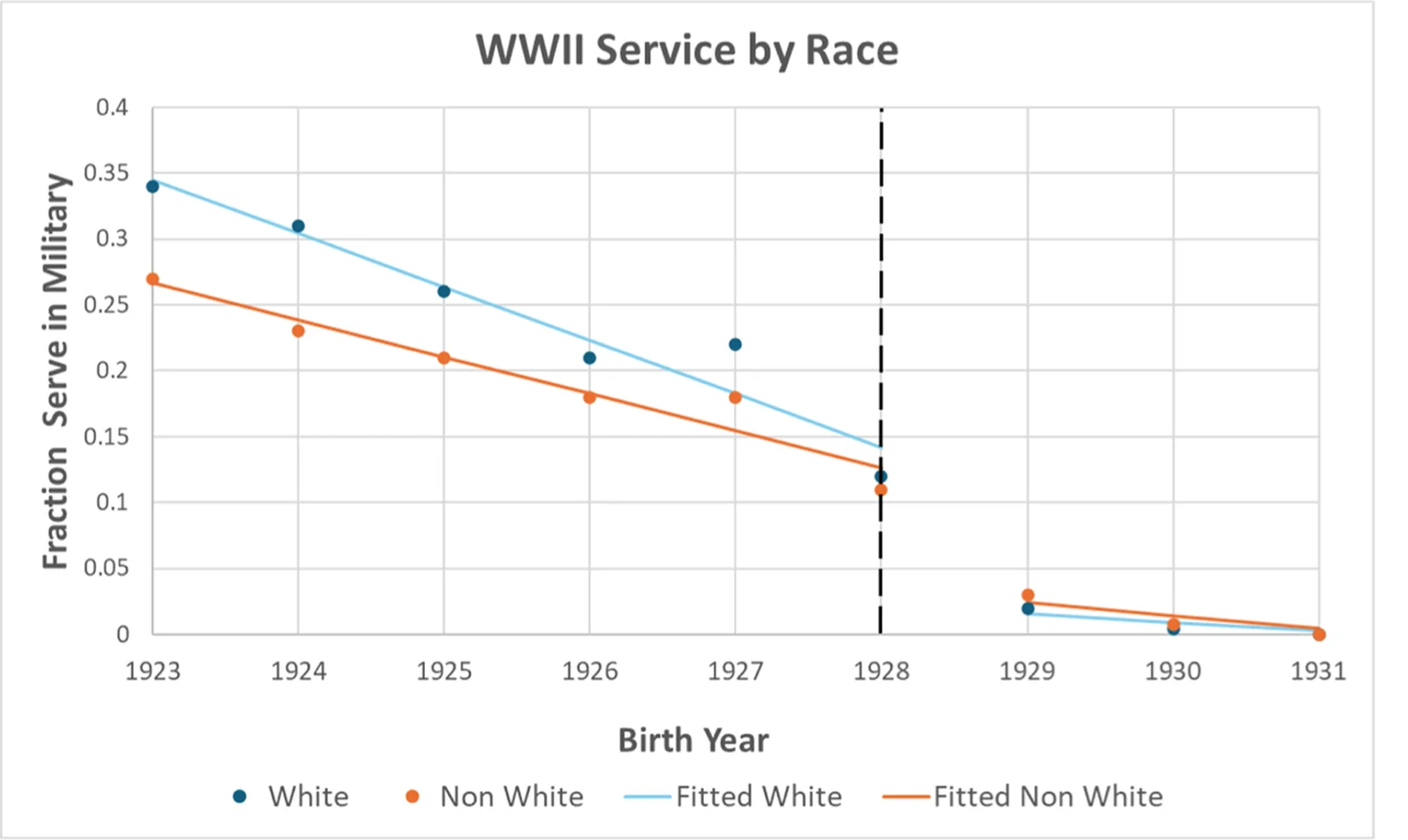
Share of WWII Army veterans by birth year among Non-Hispanic White and men from the rest of the racial and ethnic groups. Note: Graph showing fraction of White Non-Hispanic and a Non-White group including Hispanics, Asians, and Native Americans that served in WW2 by birth year cohort relative to 1928. Note: The data presented in this table are approved for dissemination by the U.S. Census Disclosure Review Board (CBDRB-FY23-CES014–052, CBDRB-FY23-CES005–028, CBDRB-FY24-CES010–007, CBRDB-FY24-CES023–014, CBDRB-FY24-CES023–016).

**Fig. 2.**
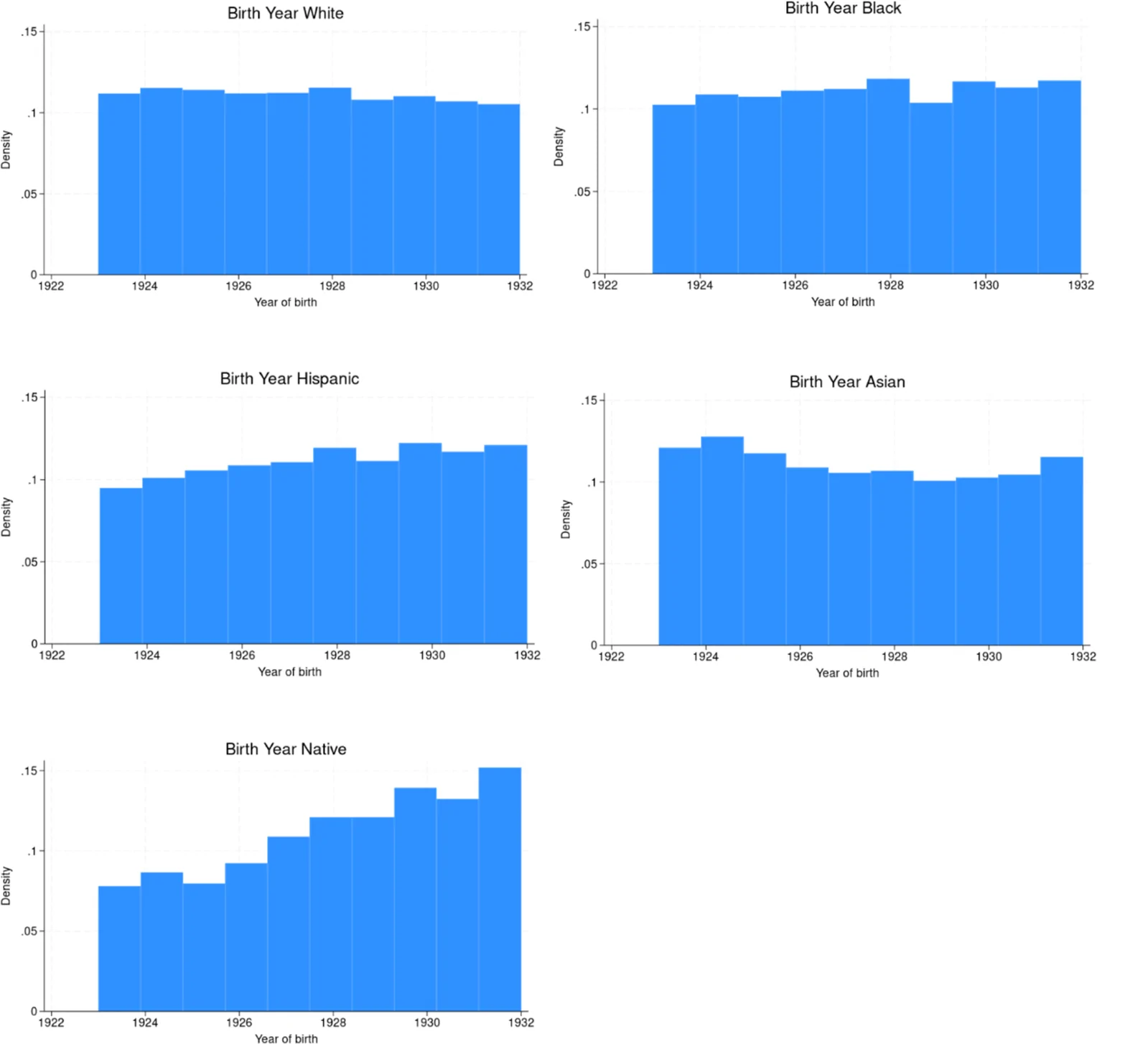
Distribution of birth year by racial group. Note: Graph distribution of birth year for each group Note: The data presented in this table are approved for dissemination by the U.S. Census Disclosure Review Board (CBDRB-FY23-CES014–052, CBDRB-FY23-CES005–028, CBDRB-FY24-CES010–007, CBRDB-FY24-CES023–014, CBDRB-FY24-CES023–016).

**Fig. 3.**
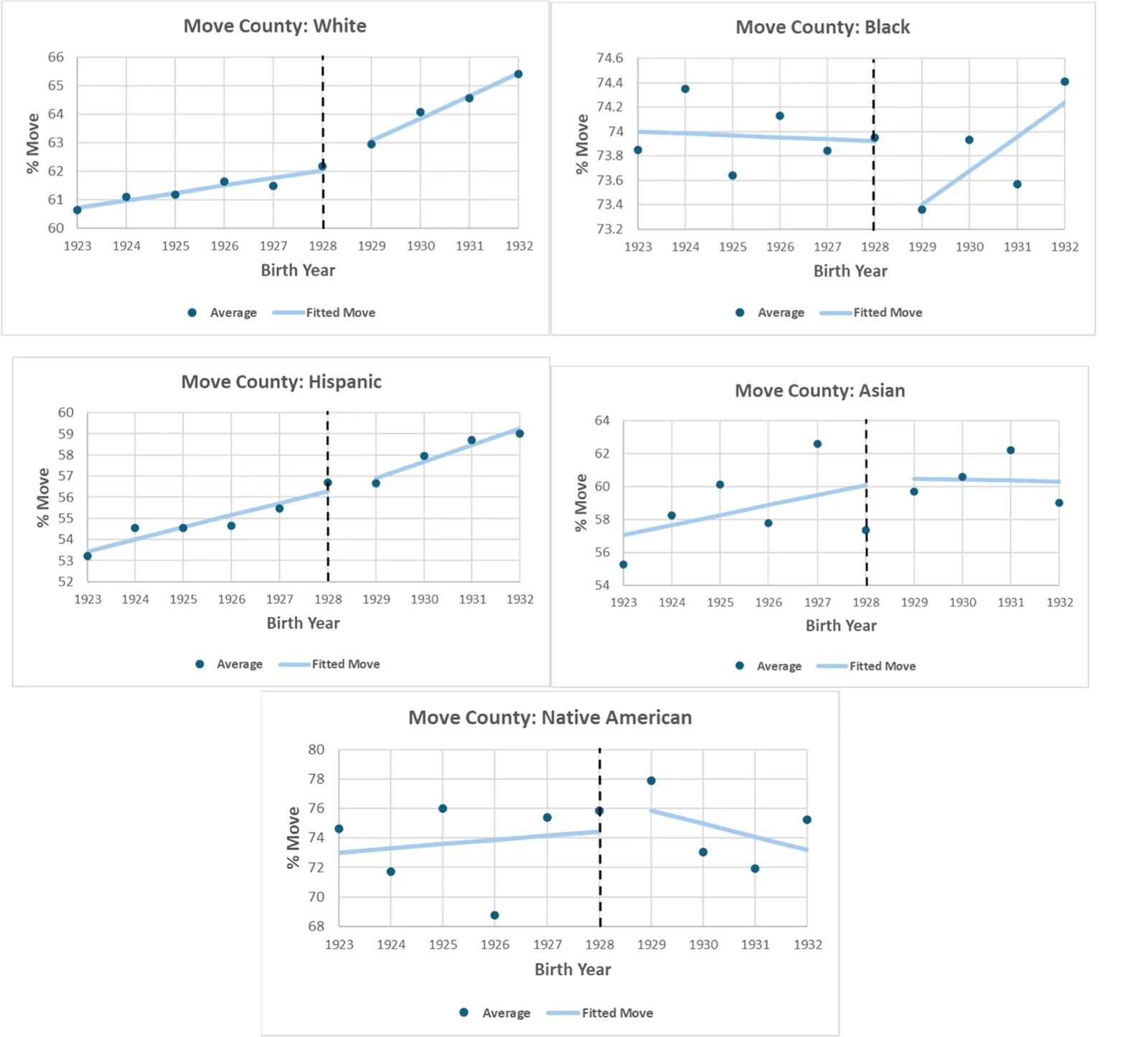
Ever move by race and birth year. Note: Graph showing fraction of individuals who moved across counties between 1940 and 1969 by race and year of birth. Note: The data presented in this table are approved for dissemination by the U.S. Census Disclosure Review Board (CBDRB-FY23-CES014–052, CBDRB-FY23-CES005–028, CBDRB-FY24-CES010–007, CBRDB-FY24-CES023–014, CBDRB-FY24-CES023–016).

**Table 1:** Summary statistics for 1940 correlates for World War II Army veterans and non-Army men of all races.

Variables	Army Veteran	Non-Army	t-statistic

Year of birth	1925	1928	−863.3***
Married by 1940	0.002	0.001	15.28***
Years of School 1940	8.2	5.5	820***
Not in Labor Force 1940	88.7	95.1	−141.5***
Per Capita HH Income 1940	250.4	238.8	28.46***
US Citizen	99.5	99.7	−17.6***
Urban 1940	0.55	0.54	24.52***
Not Moved (5yrs County) 1940	0.84	0.81	49.47***
Non-Hispanic White	0.94	0.92	45.08***
South	0.23	0.25	−36.23***
West	0.11	0.11	7.24***
Northeast	0.37	0.29	100***
Midwest	0.30	0.35	−70.71***
Observations	457,000	2,673,000	

**Note:** This table reports the mean values of the independent and dependent variables grouped by veteran status. T-statistics for the differences in means are reported in the third column assuming unequal variances. The data presented in this table are approved for dissemination by the U.S. Census Disclosure Review Board (CBDRB-FY23-CES014–052, CBDRB-FY23-CES005–028, CBDRB-FY24-CES010–007, CBRDB-FY24-CES023–014, CBDRB-FY24-CES023–016).

*** p < 0.01

** p < 0.05

* p < 0.1.

**Table 2:** Summary statistics: 1969 outcomes for World War II Army veterans and non-Army men of all races.

Variables	Veteran	Non-Veteran	t-statistic

Different County in 1940 and 1968	0.603	0.635	−41.3***
Wage and Salary Income	12400	11900	34.54***
Interest and Dividends	385.5	293.6	16.3***
Adjusted Gross Income	14500	13800	29.97***
Median Wage of Tract	6930	7010	−24.83***
Pct: Same Race/Ethnicity residents in Tract	69.9	69.7	7.79***
County Dissimilarity Index	0.225	0.234	−24.52***
Observations	457,000	2,673,000	

**Note:** Displays mean values of the independent and dependent variables grouped by veteran status. T-statistics are reported in the third column assuming unequal variances. The data presented in this table are approved for dissemination by the U.S. Census Disclosure Review Board (CBDRB- FY23-CES014–052, CBDRB-FY23-CES005–028, CBDRB-FY24-CES010–007, CBRDB-FY24-CES023–014, CBDRB-FY24-CES023–016).

*** p < 0.01

** p < 0.05

* p < 0.1.

**Table 3:** OLS estimates for differences between minority non-Army men and Non-Hispanic White non-Army men.

Outcome	Black	Hispanic	Asian	Native American

Filed 1969 Tax Return	−0.229*	−0.104*	−0.106*	−0.132*
Had Positive Interest or Dividends	−0.272*	−0.175*	0.131*	−0.181*
Had Positive Business or Farm Income	−0.157*	−0.097*	0.102*	−0.084*
Ln(Wage and Salary Income)	−0.194*	−0.162*	−0.043*	−0.196*
Ln(Interest/Dividend Income)	−0.496*	−0.298*	−0.504*	−0.293*
Ln(Business/Farm Income)	−1.042*	−0.687*	0.095	−0.311*
Ln(Adjusted Gross Income)	−0.253*	−0.211*	−0.011	−0.222*
Different County in 1940 and 1969	0.040*	−0.060*	0.015	0.009
Different State in 1940 and 1969	0.155*	0.016	0.046	−0.019
Different District in 1940 and 1969	0.166*	0.026	0.051*	−0.005
Different Region in 1940 and 1969	0.204*	0.028	0.052*	0.006
Median Wage in Census Tract	−1511*	−803*	−304*	−534*
Pct. In Tract Same Race	−0.066*	−0.475*	−0.582*	−0.663*
County Dissimilarity Index	0.095*	0.060*	0.039*	0.004*

**Notes.** Ordinary Least Squares Estimates from regression of outcome on World War II Service dummy and World War II Service interacted with minority group. The coefficients are the OLS equivalents of *β_b_* in equation 1, which is the coefficient on the minority group dummy variable. All regressions control for state of residence, year of birth, school enrollment status, years of schooling, household income per capita, employment, labor force participation, urban/rural residence, citizenship, marital status, indicator for having moved between 1935–1940, and trends for different birth years. Underlying results are reported in Appendix Tables 2–2 to 2–8. Note: The data presented in this table are approved for dissemination by the U.S. Census Disclosure Review Board (CBDRB-FY23-CES014–052, CBDRB-FY23-CES005–028, CBDRB-FY24-CES010–007, CBRDB-FY24-CES023–014, CBDRB-FY24-CES023–016). Robust standard errors were estimated

* is statistically significant in two-tailed test at the 10 % level.

**Table 4:** OLS estimates of difference between Army veterans and non-Army men within the ethnic group in the column heading.

Outcome		Non-Hispanic White	Black	Hispanic	Asian	Native American

Filed 1969 Tax Return		0.025*	0.108*	0.094*	0.116*	0.038*
Had Positive Interest or Dividends		0.011*	0.003	0.006	0.043*	0.012
Had Positive Business or Farm Income		0.005*	−0.002*	0.002	0.030*	0.009
Ln(Wage and Salary Income)		0.007*	0.043*	0.022*	0.038	0.031
Ln(Interest/Dividend Income)		0.029*	−0.081*	−0.014	−0.02	0.070
Ln(Business/Farm Income)		0.020	0.166*	0.079	0.151*	−0.032
Ln(Adjusted Gross Income)		0.012*	0.046*	0.018	0.063*	0.019
Different County in 1940 and 1969		−0.017*	−0.035*	−0.039*	0.001	−0.005
Different State in 1940 and 1969		−0.012*	−0.029*	−0.023*	0.024	0.041*
Different District in 1940 and 1969		−0.008*	−0.019*	−0.025*	0.020*	0.058*
Different Region in 1940 and 1969		−0.006*	−0.025*	−0.024*	0.003	0.045*
Median Wage in Census Tract		−26*	96*	150*	104	12
Own Wage % Diff. from Tract Median		0.014	0.019	−0.037	0.017	0.086
Pct. In Tract Same Race		0.002*	−0.003	−0.008	0.009	0.005
County Dissimilarity Index		−0.004*	0.004	−0.006*	−0.001	−0.005
MOVER (M) AND STAYER (S) DATASETS						
Had Positive Interest or Dividends	M	0.014	0.012	0.023	0.063	0.015
Had Positive Interest or Dividends	S	0.010	−0.004	−0.011	0.013	0.001
Had Positive Business or Farm Income	M	0.005	−0.001	0.007	0.043	0.018
Had Positive Business or Farm Income	S	0.006	−0.002	−0.002	0.011	−0.022
Ln(Wage and Salary Income)	M	0.013*	0.054*	0.036*	0.035	0.038
Ln(Wage and Salary Income)	S	0.006*	0.039*	0.009	0.044*	0.006
Ln(Interest/Dividend Income)	M	0.033*	−0.086*	0.036	−0.026	0.048
Ln(Interest/Dividend Income)	S	0.027*	−0.047	−0.058	−0.008	0.118
Ln(Business/Farm Income)	M	0.009	0.155*	0.249*	0.163*	0.095
Ln(Business/Farm Income)	S	0.044	0.199*	−0.092*	0.138	−0.416
Ln(Adjusted Gross Income)	M	0.017*	0.057*	0.040*	0.063*	0.025
Ln(Adjusted Gross Income)	S	0.013*	0.044*	−0.001	0.063*	−0.004
Median Wage in Census Tract	M	3	146*	192*	95	54
Median Wage in Census Tract	S	−29*	76*	142*	124	−110
Pct. In Tract Same Race	M	0.002*	0.000	−0.017	0.006	0.003
Pct. In Tract Same Race	S	0.003*	−0.009	−0.008	0.011	0.010
County Dissimilarity Index	M	−0.001	0.006	−0.004	0.002	−0.008
County Dissimilarity Index	S	−0.009*	0.029	−0.015*	−0.005	0.005

**Notes.** Ordinary Least Squares Estimates from regression of outcome on World War II Service dummy and World War II Service interacted with minority group. The coefficient for Non-Hispanic White is the OLS equivalent of *β_v_* in equation 1 and the coefficients for the minority groups are the OLS equivalent of the sum of *β_v_* and *β_vb_* in equation 1 for each of the minority groups. All regressions control for state of residence, year of birth, school enrollment status, years of schooling, household income per capita, employment, labor force participation, urban/rural residence, citizenship, marital status, indicator for having moved between 1935–1940, and trends for different birth years. Underlying results are in Appendix Tables 2–2 to 2–8. Note: The data presented in this table are approved for dissemination by the U.S. Census Disclosure Review Board (CBDRB-FY23-CES014–052, CBDRB-FY23-CES005–028, CBDRB-FY24-CES010–007, CBRDB-FY24-CES023–014, CBDRB-FY24-CES023–016). Robust standard errors were estimated.

* is statistically significant in two-tailed test at the 10 % level.

**Table 5:** FRD first stage results in pooled sample with World War II army service interacted by group.

Variables	(1) Army	(2) Black x Army	(3) Hispanic x Army	(4) Asian x Army	(5) Native x Army

Born ≤ 1928	0.104*** (0.000575)	−0.00022*** (1.57E-05)	−0.00011*** (9.79E-06)	−9.35e-06*** (2.58E-06)	1.93E-06 (2.04E-06)
Black x Born ≤ 1928	−0.0383*** (0.00227)	0.0664*** (0.00219)	1.48E-05 (2.01E-05)	1.95E-06 (2.35E-06)	−9.98E-06 (9.51E-06)
Hispanic x Born ≤ 1928	−0.00466 (0.00447)	−0.000197*** (5.5E-05)	0.102*** (0.00442)	−8.86E-06 (5.40E-06)	1.9E-06 (3.03E-06)
Asian x Born ≤ 1928	0.0683*** (0.0116)	0.00014 (0.00012)	0.000105 (0.000153)	0.173*** (0.0116)	1.97E-07 (4.46E-06)
Native x Born ≤ 1928	−0.0181 (0.0139)	−0.000291 (0.00039)	2.95E-05 (0.000218)	2.74E-06 (1.95E-05)	0.0859*** (0.0138)
Observations	3125000	3125000	3125000	3125000	3125000
R squared	0.139	0.167	0.221	0.311	0.183
Instrument F-stat	6830	220	130	47.1	8.18

**Notes:** Fuzzy Regression Discontinuity Estimates from First Stage Regression, regressing World War II Service on cutoff variable and World War II Service interacted with minority group and cutoff variable interacted with minority group. All regressions control for state of residence, year of birth, school enrollment status, years of schooling, household income per capita, employment, labor force participation, urban/rural residence, citizenship, marital status, indicator for having moved between 1935–1940, and race specific running variable. Note: The data presented in this table are approved for dissemination by the U.S. Census Disclosure Review Board (CBDRB-FY23-CES014–052, CBDRB-FY23-CES005–028, CBDRB-FY24-CES010–007, CBRDB-FY24-CES023–014, CBDRB-FY24-CES023–016, CBDRB-FY25-CES010–002). Robust standard errors in parentheses

*** p < 0.01

** p < 0.05

* p < 0.1.

**Table 6:** FRD estimates of differences in outcomes between Army veterans and non-Army men within the same group.

Outcome		Non-Hispanic White	Black	Hispanic	Asian	Native American

Filed 1969 Tax Return		−0.079*	−0.081	−0.022	−0.067	−0.196
Had Positive Interest or Dividends		−0.070*	−0.048	−0.051	0.176	−0.347
Had Positive Business or Farm Income		−0.016*	0.01	−0.183*	0.256*	−0.213
Ln(Wage and Salary Income)		−0.038*	−0.182*	0.044	−0.262	−0.054
Ln(Interest/Dividend Income)		−0.186*	−0.232	−0.963*	−0.197	3.188
Ln(Business/Farm Income)		−0.343*	−2.135*	−0.174	−0.332	−0.201
Ln(Adjusted Gross Income)		−0.079*	−0.325*	0.008	−0.057	0.041
Different County in 1940 and 1969		−0.051*	−0.004	0.018	−0.049	−0.156
Different State in 1940 and 1969		−0.112*	0.026	−0.061	−0.004	0.159
Different District in 1940 and 1969		−0.121*	0.009	−0.073	−0.008	0.293
Different Region in 1940 and 1969		−0.1*	0.077	−0.033	0.021	0.391
Median Wage in Census Tract		−385*	−159	−188	−945	−2069
Pct. In Tract Same Race		−0.006*	−0.034	−0.024	−0.025	0.024
County Dissimilarity Index		−0.005	−0.022	−0.001	−0.049	−0.181*
MOVER (M) AND STAYER (S) DATASETS						
Had Positive Interest or Dividends	M	−0.06*	−0.024	−0.042	0.067	−0.429
Had Positive Interest or Dividends	S	−0.088*	−0.124	−0.064	0.343	0.047
Had Positive Business or Farm Income	M	0.005	−0.07	−0.197	0.171	0.124
Had Positive Business or Farm Income	S	−0.055*	0.200*	−0.169	0.391	0.49
Ln(Wage and Salary Income)	M	−0.042	−0.166	−0.106	−0.226	0.215
Ln(Wage and Salary Income)	S	−0.033	−0.233	0.19	−0.363	−0.9
Ln(Interest/Dividend Income)	M	−0.088	−0.076	−1.359*	−0.112	2.385
Ln(Interest/Dividend Income)	S	−0.351*	−0.736	−0.539	−0.404	7.477
Ln(Business/Farm Income)	M	−0.215*	−1.414	0.814	0.21	−0.17
Ln(Business/Farm Income)	S	−0.573*	−3.91*	−1.35	−1.501	−2.088
Ln(Adjusted Gross Income)	M	−0.047*	−0.298*	−0.114	−0.048	0.218
Ln(Adjusted Gross Income)	S	−0.113*	−0.418*	0.126	−0.118	−0.551
Median Wage in Census Tract	M	−343*	−201	−43	−1490*	−1896
Median Wage in Census Tract	S	−315*	−22	−386	−173	−2097
Pct. In Tract Same Race	M	−0.001	−0.024	−0.049	−0.018	0.096
Pct. In Tract Same Race	S	−0.013*	−0.061	0.002	−0.031	−0.281
County Dissimilarity Index	M	−0.006	−0.013	0.014	−0.064	−0.124
County Dissimilarity Index	S	0.005	−0.059	−0.013	−0.031	−0.37

**Notes:** Fuzzy Regression Discontinuity estimates of impact of WWII service on outcomes within each group. Coefficients in each row come from regression for that outcome. Non-Hispanic White value is estimate of *β_v_* and value for Blacks is sum of *β_v_* and *β_vb_* in equation 1 in text with coefficients for other minorities following pattern for Blacks. All regressions control for state of residence, year of birth, school enrollment status, years of schooling, household income per capita, employment, labor force participation, urban/rural residence, citizenship, marital status indicator for having moved between 1935–1940, and race specific running variable. Results from regressions with standard errors are reported in Appendix Tables 1–1 through 1–7. Note: The data presented in this table are approved for dissemination by the U.S. Census Disclosure Review Board (CBDRB-FY23-CES014–052, CBDRB-FY23-CES005–028, CBDRB-FY24-CES010–007, CBRDB-FY24-CES023–014, CBDRB-FY24-CES023–016, CBDRB-FY25-CES010– 002). Robust standard errors were estimated

* is statistically significant in two-tailed test at the 10 % level.

**Table 7:** FRD difference-in-difference estimates: minority group Army/non-Army difference minus Non-Hispanic White Army/non-Army difference.

Outcome		Black	Hispanic	Asian	Native American

Filed 1969 Tax Return		−0.002	0.057	0.011	−0.117
Had Positive Interest or Dividends		0.023	0.019*	0.246*	−0.276
Had Positive Business or Farm Income		0.025	−0.167*	0.271*	−0.229
Ln(Wage and Salary Income)		−0.144	0.082	−0.224	−0.015
Ln(Interest/Dividend Income)		−0.046	−0.777*	−0.011	3.374
Ln(Business/Farm Income)		−1.790*	0.170	0.011	0.142
Ln(Adjusted Gross Income)		−0.246*	0.087	0.022	0.12
Different County in 1940 and 1969		0.047	0.069	0.003	−0.104
Different State in 1940 and 1969		0.138*	0.051	0.107	0.271
Different District in 1940 and 1969		0.130*	0.048	0.113	0.415
Different Region in 1940 and 1969		0.178*	0.068	0.121	0.492
Median Wage in Census Tract		226	197	560	−1682
Pct. In Tract Same Race		−0.028	−0.018	−0.019	0.031
County Dissimilarity Index		−0.018	0.004	−0.044	−0.176*
MOVER (M) AND STAYER (S) DATASETS					
Had Positive Interest or Dividends	M	0.036	0.018	0.127	−0.369
Had Positive Interest or Dividends	S	−0.036	0.024	0.431*	0.135
Had Positive Business or Farm Income	M	−0.075	−0.202	0.166	0.119
Had Positive Business or Farm Income	S	−0.256*	−0.113	0.446*	0.545
Ln(Wage and Salary Income)	M	−0.141	−0.081	−0.201	0.241
Ln(Wage and Salary Income)	S	−0.200	0.224	−0.330	−0.867
Ln(Interest/Dividend Income)	M	0.012	−1.271*	−0.024	2.473
Ln(Interest/Dividend Income)	S	−0.385	−0.188	−0.053	7.828
Ln(Business/Farm Income)	M	−1.199	1.029	0.425	0.045
Ln(Business/Farm Income)	S	−3.337*	−0.777	−0.929	−1.515
Ln(Adjusted Gross Income)	M	−0.251*	−0.067	−0.001	0.265
Ln(Adjusted Gross Income)	S	−0.305	0.239*	−0.005	−0.439
Median Wage in Census Tract	M	142	299	−1147	−1552
Median Wage in Census Tract	S	292	−72	141	−1783
Pct. In Tract Same Race	M	−0.023	−0.048	−0.017	0.098
Pct. In Tract Same Race	S	−0.047	0.016	−0.018	−0.268
County Dissimilarity Index	M	−0.007	0.02	−0.058	−0.117
County Dissimilarity Index	S	−0.052	−0.008	−0.026	−0.365

**Notes:** Fuzzy Regression Discontinuity Estimates of impact of WWII service on outcomes within each group. Coefficients in each column row come from regression for that outcome. Black value is *β_vb_* in equation 1, which reflects the veteran/non-veteran difference for Blacks minus the veteran/ non-veteran difference for Non-Hispanic White men. Values for other minorities are coefficient on the interaction of minority dummy with WWII service prediction. All regressions control for state of residence in 1969, year of birth, and measures from 1940 Census on education, household income per capita, urban/rural residence, citizenship, marital status, indicator for having moved between 1935–1940, and race specific running variable. Underlying results are in Appendix Tables 1–1 through 1–7. Note: The data presented in this table are approved for dissemination by the U.S. Census Disclosure Review Board (CBDRB-FY23-CES014–052, CBDRB-FY23-CES005–028, CBDRB-FY24-CES010–007, CBRDB-FY24-CES023–014, CBDRB-FY24-CES023–016). Robust standard errors were estimated

* is statistically significant in two-tailed test at the 10 % level.

**Table 8:** OLS and IV results for the natural log of wage and salary income of non-Hispanic White males in 1959 using different definitions of military service from the IPUMS 5 % sample.

	OLS For Men Born 1923–1932			IV For Men Born 1923–1932		
	1	2	3	4	5	6

	Army 70 % of WWII Alone	WWII Alone	WWII and Korea	Army 70 % of WWII Alone	WWII Alone	WWII and Korea
Army	0.120 (46.18)			−0.040 (−2.10)		
WWII		0.202 (64.81)	0.218 (68.41)		−0.028 (−2.10)	0.008 (0.57)
Korea			0.091 (33.50)			0.059 (16.41)
Run	Yes	Yes	Yes	Yes	Yes	Yes
Run*born 1929–1932	Yes	Yes	Yes	Yes	Yes	Yes
Birthplace Fixed Effects	Yes	Yes	Yes	Yes	Yes	Yes
Number of Observations	373,275	373,275	373,275	373,275	373,275	373,275
R-squared	0.049	0.056	0.059	0.040	0.040	0.046

**Notes:** Presents results evaluating the effect of omitting a portion of World War II sample on main results using the 1960 5 % Census from IPUMS. Columns (1) and (4) randomly only assigns 70 % of the veteran sample to the variable titled Army while leaving the rest in the control group. Column (2) and (5) assigns all World War II veterans to veteran variable called WWII, while column (3) and (6) use the WWII variable with all veterans assigned while controlling for Korean War service. Source: Ruggles et al., 2024b.

## Data Availability

The data that has been used is confidential.

## Footnotes

1 Asians include Chinese, Japanese, and Pacific Islanders. When we separated out the Japanese observations, many of the statistically significant results are the same. We lose precision and the magnitudes of some results rise.

2 These two strands of the literature are well-developed and while we reference further work in the next sections, it would be beyond the scope of the paper to provide a comprehensive survey. Ferrara (2023) surveys the literature on the labor market effects of WWII for women and Black Americans.

3 For Hispanics, the number is estimated to be between 400,000 and 500,000 service members because official statistics did not treat them as separate group in racial breakdowns and instead counted them as white soldiers.

4 The typical draft board had three members or more. Most counties tended to have one board, especially in rural areas, but could have substantially more in denser urban places and cities.

5 About 1.1 % and 12.3 % of white and Black draftees, respectively, were rejected for “insufficient education” between May 15 and September 15, 1941. In 1943, around 33 % of White draftees were rejected for any reason compared to a rejection rate of 50 % among Black draftees.

6 Most notably, the “Buffalo soldiers” of the 92^nd^ Infantry Division, the “Black Panthers” of the 761^st^ Anti-Tank Battalion, and the Tuskegee Airmen, among others, quickly rose to fame.

7 For a recent review, see Ferrara (2023).

8 Angrist and Krueger (1994) even document a wage loss for veterans compared to non-veterans after controlling for selection into the military.

9 The effects of service on wages and other later-life economic outcomes are ambiguous a priori.

10 We based the census tracts on the 2010 boundaries. There were changes in census tracts after 1969, so some observations are not assigned to a tract. We examined the income results with and without the observations that were missing the census tracts, and the results were very similar.

11 The dissimilarity index is a measure of segregation that focuses on 2 groups, whites and non-whites. It measures the minimum percentage of non-whites in the county who would have to move so that the non-whites and whites are spread evenly among the census tracts in the county. For each census tract in the county calculate tract *i’*s share of the county white population, $W_{i}$, and tract *i*’s share of the county non-white population, $N_{i}$. If the non-white tract share is greater than the white tract share $\left(N_{i}>W_{i}\right)$ , a dummy variable $D_{i}$ is set equal to 1; otherwise, it is zero. The dissimilarity index is the sum across all tracts in the county of the dummy multiplied by the difference between the tract’s share of county non-whites and the tract’s share of county whites, $DI=\sum D_{i}*(N_{i}-W)$.

12 Massey et al. (2018) describes the method for assigning PIKs. The match rate for the beta version of the PIKs with the 1940 census as of 2018 was between 60 and 70 percent for the age group we examine. The match rates for the PIKs with the Military and IRS records are above 95 percent.

13 The low rate of non-citizenship could be explained by the age range of our sample in 1940, 8- to 17-year-olds as well as the fact that the 1920s and the 1930s witnessed major restrictions on immigration in the 1920s Quota’s act and the 1930 Mexican Repatriations.

14 The filing of birth certificates improved as states joined the Birth Registration Area between 1910 and 1933, but the Census Bureau comparisons between birth certificates and lists of births from a variety of sources found match rates of 94 percent for whites and 82 percent for blacks (Eriksson et al., 2018, p.2006).

15 We included state fixed effects for residence in 1940, while they included county fixed effects in 1940 and state fixed effects for the state of birth. Our education variables are the highest grade for the young man and school attendance, while they included the highest grade and school attendance of the young man, the father’s highest grade and a dummy for the father’s college completion. For birth year, we used the running variable structure for birth year, while they used birth year fixed effects. For family resources, we used household wage and salary income per capita, while they used father’s wage and salary income, occupational score, and dummies for whether the father was in agriculture, in manufacturing, or was in the following skill category: white collar, unskilled, farmer, craft, and operatives. We both included urban status, and they added farm status. We included a migration and citizenship measures that they did not include. They included home ownership, which we did not include. They included group-specific state fixed effects and group-specific county fixed effects, and we did not.

16 When we estimate the OLS models without controls, the Army/non-Army difference in incomes is somewhat more positive than the results with controls in Table 4, which is consistent with positive selection related to the correlates include in the regressions for Table 4. The comparisons are similar to what Collins and Zimran (2025) found.

17 The estimates for White men look very similar to estimates in a regression with all groups without any of the group dummies or interactions because Whites account for more than 90 percent of the dataset.

18 Information downloaded on 12/13/2024 from https://www.armydivs.com/#:~:text=During%20World%20War%20II%20about,660 %2C000 %20served%20in%20the%20Marines.
