# Supplementary material for: The impact of World War II Army service on income and mobility in the 1960s by ethnoracial group
Source: Explor Econ Hist. Author manuscript; Available in PMC 2026 Oct 20. (PMC13679824; doi:10.1016/j.eeh.2025.101687)
Supplement: Supplementary file 1 [file NIHMS2129177-supplement-supplementary_materials.docx]

**Online Appendix for**

***The Impact of World War II Army Service on Income and Mobility in the 1960s by Ethnoracial Group***

Sergio E. Barrera^[1]^*	Andreas Ferrara^[2]†^	Price V. Fishback^[3]‡^	Misty L. Heggeness^[4]§^

**April 15, 2025**

|  | **APPENDIX OF FUZZY REGRESSION DISCONTINUITY RESULTS**  **Appendix Table 1-1: FRD Results for Filing Tax Return and Reporting Interest/Dividend and Business/Farm Income** | | | | | | |
| --- | --- | --- | --- | --- | --- | --- | --- |
|  | | (1) | | (2) | | (3) | |
| VARIABLES | | Filed 1040 | | Positive Interest or Dividend Income | | Positive Business or Farm Income | |
|  | |  | |  | |  | |
| ARMY VETERAN COMPARISON WITH NON-ARMY MEN AMONG NON-HISPANIC WHITES | | | | | | | |
| NH White WWII | | -0.0787*** | | -0.0704*** | | -0.0156 | |
|  | | (0.00591) | | (0.0125) | | (0.0126) | |
| DIFFERENCE-IN-DIFFERENCE BETWEEN MINORITY ARMY EFFECT AND NON-HISPANIC WHITE ARMY EFFECT | | | | | | | |
| Black x WWII | | -0.00234 | | 0.0227 | | 0.0251 | |
|  | | (0.0615) | | (0.0658) | | (0.0678) | |
| Hispanic x WWII | | 0.0569 | | 0.0193 | | -0.167** | |
|  | | (0.0636) | | (0.0833) | | (0.0825) | |
| Asian x WWII | | 0.0113 | | 0.246* | | 0.271* | |
|  | | (0.0997) | | (0.128) | | (0.143) | |
| Native x WWII | | -0.117 | | -0.276 | | 0.229 | |
|  | | (0.194) | | (0.329) | | (0.326) | |
|  | |  | |  | |  | |
| Mean | | 0.861 | | 0.561 | | 0.407 | |
| Observations | | 5928000 | | 3125000 | | 3125000 | |
| Weak Instruments F | | 349.1 | | 183 | | 183 | |
| **Notes:** Fuzzy Regression Discontinuity Estimates of impact of WWII Army service on outcomes within each group. Coefficients in each column come from regression for that outcome. White value is equation 1 estimate of *β_v_*, which reflects the difference in outcome between White Army veterans and White non-Army men. Black value is *β_vb_* in equation 1, which reflects the Army/non-Army difference for Blacks minus the Army/non-Army difference for White men. The minority values are like the black values. All regressions control for state of residence, year of birth, school enrollment status, years of schooling, household income per capita, urban/rural residence, citizenship, marital status, indicator for having moved between 1935-1940, and race specific running variable. Note: The data presented in this table are approved for dissemination by the U.S. Census Disclosure Review Board (CBDRB-FY23-CES014-052, CBDRB-FY23-CES005-028, CBDRB-FY24-CES010-007, CBRDB-FY24-CES023-014, CBDRB-FY24-CES023-016). Robust standard errors in parentheses. *** p*<*0.01, ** p*<*0.05, * p*<*0.1. | | | | | | | |
|  | **Appendix Table 1-2: FRD Results for Natural Log of Incomes for Filers Who Reported Positive Amounts** | | | | | | |
|  |  | (1) | (2) | | (3) | | (4) |
|  | VARIABLES | Ln(Wage & Salary) | Ln(Interest & Dividends) | | Ln(Business &  Farm) | | Ln(Adjusted Gross Income) |
|  |  |  |  | |  | |  |
|  | ARMY VETERAN COMPARISON WITH NON-ARMY MEN AMONG NON-HISPANIC WHITES | | | | | | |
|  | WWII | -0.0382** | -0.186*** | | -0.343*** | | -0.0791*** |
|  |  | (0.0176) | (0.0519) | | (0.101) | | (0.0144) |
|  | DIFFERENCE-IN-DIFFERENCE BETWEEN MINORITY ARMY EFFECT AND NON-HISPANIC WHITE ARMY EFFECT | | | | | | |
|  | Black x WWII | -0.144 | -0.0457 | | -1.79** | | -0.246** |
|  |  | (0.11) | (0.362) | | (0.786) | | (0.108) |
|  | Hispanic x WWII | 0.0818 | -0.777* | | 0.17 | | 0.0869 |
|  |  | (0.117) | (0.404) | | (0.838) | | (0.107) |
|  | Asian x WWII | -0.224 | -0.0109 | | 0.0114 | | 0.0218 |
|  |  | (0.216) | (0.458) | | (0.927) | | (0.166) |
|  | Native x WWII | -0.0154 | 3.374 | | 0.142 | | 0.12 |
|  |  | (0.518) | (2.267) | | (3.372) | | (0.482) |
|  |  |  |  | |  | |  |
|  | Mean | 9.286 | 4.795 | | 5.874 | | 9.372 |
|  | Observations | 2953000 | 1753000 | | 1271000 | | 3115000 |
|  | Weak Instruments F | 180 | 2.58 | | 49.6 | | 182 |
|  | **Notes.** See notes to Appendix Table 1-1. The data presented in this table are approved for dissemination by the U.S. Census Disclosure Review Board (CBDRB-FY23-CES014-052, CBDRB-FY23-CES005-028, CBDRB-FY24-CES010-007, CBRDB-FY24-CES023-014, CBDRB-FY24-CES023-016). | | | | | | |

|  | **Appendix Table 1-3: FRD Results for Differences in Location Between 1969 and 1940** | | | | | |
| --- | --- | --- | --- | --- | --- | --- |
|  |  | (1) | (2) | (3) | (4) | |
|  | VARIABLES | Different County | Different State | Different District | Different Region | |
|  |  |  |  |  |  | |
|  | ARMY VETERAN COMPARISON WITH NON-ARMY MEN AMONG NON-HISPANIC WHITES | | | | | |
|  | WWII | -0.0513*** | -0.112*** | -0.121*** | -0.100*** | |
|  |  | (0.0119) | (0.0117) | (0.0108) | (0.0101) | |
| DIFFERENCE-IN-DIFFERENCE BETWEEN MINORITY ARMY EFFECT AND NON-HISPANIC WHITE ARMY EFFECT | | | | | |  |
|  | Black x WWII | 0.0472 | 0.138* | 0.130* | 0.178** | |
|  |  | (0.0659) | (0.0765) | (0.0763) | (0.0782) | |
|  | Hispanic x WWII | 0.069 | 0.0514 | 0.0482 | 0.0677 | |
|  |  | (0.0842) | (0.0749) | (0.0726) | (0.0635) | |
|  | Asian x WWII | 0.0026 | 0.107 | 0.113 | 0.121 | |
|  |  | (0.133) | (0.112) | (0.106) | (0.0953) | |
|  | Native x WWII | -0.104 | 0.271 | 0.415 | 0.492 | |
|  |  | (0.293) | (0.335) | (0.321) | (0.304) | |
|  | Mean | 0.63 | 0.352 | 0.263 | 0.212 | |
|  | Observations | 3125000 | 3125000 | 3125000 | 3125000 | |
|  | Weak Instruments F | 183 | 183 | 183 | 183 | |
|  | **Notes.** See notes to Appendix Table 1-1. District refers to 9 Census Districts, e.g. South Atlantic. Region refers to 4 Census Regions, e.g. South. The data presented in this table are approved for dissemination by the U.S. Census Disclosure Review Board (CBDRB-FY23-CES014-052, CBDRB-FY23-CES005-028, CBDRB-FY24-CES010-007, CBRDB-FY24-CES023-014, CBDRB-FY24-CES023-016). | | | | | |

|  | **Appendix Table 1-4: Local Area Characteristics: Service Impacts Between Minorities and White Veterans** | | | | | | | |
| --- | --- | --- | --- | --- | --- | --- | --- | --- |
|  |  | (1) | (2) | | (3) | |  |  |
|  | VARIABLES | Tract Wage | % Same Race in Tract | | Dissimilarity Index for County | |  |  |
|  |  |  |  |  | |  | | |
|  | ARMY VETERAN COMPARISON WITH NON-ARMY MEN AMONG NON-HISPANIC WHITES | | | | | | | |
|  | WWII | -384.8*** | -0.00634** | | -0.0046 | |  |  |
|  |  | (56.64) | (0.00248) | | (0.00327) | |  |  |
| DIFFERENCE-IN-DIFFERENCE BETWEEN MINORITY ARMY EFFECT AND NON-HISPANIC WHITE ARMY EFFECT | | | | | | | |  |
|  | Black x WWII | 226 | -0.028 | | -0.0178 | |  |  |
|  |  | (245.7) | (0.0543) | | (0.0241) | |  |  |
|  | Hispanic x WWII | 196.7 | -0.018 | | 0.00373 | |  |  |
|  |  | (375.1) | (0.0408) | | (0.0221) | |  |  |
|  | Asian x WWII | -560.3 | -0.0185 | | -0.0441 | |  |  |
|  |  | (671) | (0.0426) | | (0.0319) | |  |  |
|  | Native x WWII | -1682 | 0.0307 | | -0.176* | |  |  |
|  |  | (1563) | (0.0856) | | (0.0921) | |  |  |
|  |  |  |  | |  | |  |  |
|  | Mean | 7000 | 0.698 | | 0.233 | |  |  |
|  | Observations | 3125000 | 3125000 | | 3125000 | |  |  |
|  | Weak Instruments F | 183 | 183 | | 183 | |  |  |

| **Appendix Table 1-5: FRD Results for Reporting Types of Income in Dataset for Movers and Dataset for Stayers** | | | | | | | | | | | | | | | | | |
| --- | --- | --- | --- | --- | --- | --- | --- | --- | --- | --- | --- | --- | --- | --- | --- | --- | --- |
|  | | (1) | | | (2) | | | | | | | | | | | | |
| VARIABLES | | Any Interest or Dividends | | | Any Business or Farm Income | | | | | | | | | | | | |
|  | |  | |  | | | |  | | |  | | | | | | |
| **Panel 1: Movers Dataset** | | | | | | | | | | | | | | | | | |
| ARMY VETERAN COMPARISON WITH NON-ARMY MEN AMONG NON-HISPANIC WHITES | | | | | | | | | | | | | | | | | |
| White WWII | | -0.0595*** | | | 0.00497 | | | | | | | | | | | | |
|  | | (0.0157) | | | (0.0158) | | | | | | | | | | | | |
| DIFFERENCE-IN-DIFFERENCE BETWEEN MINORITY ARMY EFFECT AND NON-HISPANIC WHITE ARMY EFFECT | | | | | | | | | | | | | | |  |  |  |
| Black WWII | | 0.036 | | | -0.0746 | | | | | | | | | | | | |
|  | | (0.0792) | | | (0.0814) | | | | | | | | | | | | |
| Hispanic WWII | | 0.017 | | | -0.202 | | | | | | | | | | | | |
|  | | (0.124) | | | (0.123) | | | | | | | | | | | | |
| Asian WWII | | 0.127 | | | 0.166 | | | | | | | | | | | | |
|  | | (0.159) | | | (0.176) | | | | | | | | | | | | |
| Native WWII | | -0.369 | | | 0.119 | | | | | | | | | | | | |
|  | | (0.363) | | | (0.353) | | | | | | | | | | | | |
| Observations | | 1969000 | | | 1969000 | | | | | | | | | | | | |
| First Stage F-stat | | 140.1 | | | 140.1 | | | | | | | | | | | | |
| **Panel 2: Stayers Dataset** | | | | | | | | | | | | | | | | | |
| ARMY VETERAN COMPARISON WITH NON-ARMY MEN AMONG NON-HISPANIC WHITES | | | | | | | | | | | | | | | | | |
| WWII | | -0.0880*** | | | -0.0552*** | | | | | | | | | | | | |
|  | | (0.0206) | | | (0.0208) | | | | | | | | | | | | |
| DIFFERENCE-IN-DIFFERENCE BETWEEN MINORITY ARMY EFFECT AND NON-HISPANIC WHITE ARMY EFFECT | | | | | | | | | | | | | | |  |  |  |
| Black x WWII | | -0.0363 | | | 0.256** | | | | | | | | | | | | |
|  | | (0.116) | | | (0.123) | | | | | | | | | | | | |
| Hispanic x WWII | | 0.0238 | | | -0.113 | | | | | | | | | | | | |
|  | | (0.112) | | | (0.109) | | | | | | | | | | | | |
| Asian x WWII | | 0.431** | | | 0.446* | | | | | | | | | | | | |
|  | | (0.214) | | | (0.244) | | | | | | | | | | | | |
| Native x WWII | | 0.135 | | | 0.545 | | | | | | | | | | | | |
|  | | (0.768) | | | (0.802) | | | | | | | | | | | | |
|  | |  | | |  |  | | | | | | | |  |  |  |  |
| Observations | | 1156000 | | | 1156000 | | | | | | | | | | | | |
| First Stage F-stat | | 44.83 | | | 44.83 | | | | | | | | | | | | |
| **Notes.** See notes to Appendix Table 1-1. The data presented in this table are approved for dissemination by the U.S. Census Disclosure Review Board (CBDRB-FY23-CES014-052, CBDRB-FY23-CES005-028, CBDRB-FY24-CES010-007, CBRDB-FY24-CES023-014, CBDRB-FY24-CES023-016). | | | | | | | | | | | | | | | | | |
|  | **Appendix Table 1-6: FRD Results for Natural Log of Income for Four Types of Income in Dataset for Movers and Dataset for Stayers** | | | | | | | | | | | | | | |  |  |
|  |  | | (1) | | | | (2) | | | (3) | | | (4) | | |  |  |
|  | VARIABLES | | Ln(Wage and Salary) | | | | Ln(Interest and Dividends) | | | Ln(Business and Farm) | | | Ln(Adjusted Gross Income) | | |  |  |
|  |  | |  | | | |  | | |  | | |  | | |  |  |
|  | **Panel 1: Dataset for Movers Who Lived in Different Counties in 1940 and 1969** | | | | | | | | | | | | | | |  |  |
|  | ARMY VETERAN COMPARISON WITH NON-ARMY MEN AMONG NON-HISPANIC WHITES | | | | | | | | | | | | | | |  |  |
|  | WWII | | -0.0252 | | | | -0.0884 | | | -0.215* | | | -0.0470*** | | |  |  |
|  |  | | (0.0218) | | | | (0.0649) | | | (0.125) | | | (0.0181) | | |  |  |
|  | DIFFERENCE-IN-DIFFERENCE BETWEEN MINORITY ARMY EFFECT AND NON-HISPANIC WHITE ARMY EFFECT | | | | | | | | | | | | | | |  |  |
|  | Black x WWII | | -0.141 | | | | 0.0123 | | | -1.199 | | | -0.251** | | |  |  |
|  |  | | (0.129) | | | | (0.421) | | | (0.915) | | | (0.127) | | |  |  |
|  | Hispanic x WWII | | -0.0809 | | | | -1.271** | | | 1.029 | | | -0.0672 | | |  |  |
|  |  | | (0.174) | | | | (0.591) | | | (1.209) | | | (0.157) | | |  |  |
|  | Asian x WWII | | -0.201 | | | | -0.0237 | | | 0.425 | | | -0.000794 | | |  |  |
|  |  | | (0.254) | | | | (0.569) | | | (1.152) | | | (0.214) | | |  |  |
|  | Native x WWII | | 0.241 | | | | 2.473 | | | 0.0449 | | | 0.265 | | |  |  |
|  |  | | (0.552) | | | | (2.341) | | | (3.524) | | | (0.525) | | |  |  |
|  | Observations | | 1869000 | | | | 1098000 | | | 785000 | | | 1963000 | | |  |  |
|  | Weak Instruments F | | 137.4 | | | | 57 | | | 39.44 | | | 138.6 | | |  |  |
|  | **Panel 2: Stayers Dataset** | | | | | | | | | | | | | | |  |  |
|  | ARMY VETERAN COMPARISON WITH NON-ARMY MEN AMONG NON-HISPANIC WHITES | | | | | | | | | | | | | | |  |  |
|  | WWII | | -0.0334 | | | | -0.351*** | | | -0.573*** | | | -0.113*** | | |  |  |
|  |  | | (0.0292) | | | | (0.0865) | | | (0.169) | | | (0.0236) | | |  |  |
|  | DIFFERENCE-IN-DIFFERENCE BETWEEN MINORITY ARMY EFFECT AND NON-HISPANIC WHITE ARMY EFFECT | | | | | | | | | | | | | | |  |  |
|  | Black x WWII | | -0.2 | | | | -0.385 | | | -3.337** | | | -0.305 | | |  |  |
|  |  | | (0.207) | | | | (0.721) | | | (1.589) | | | (0.202) | | |  |  |
|  | Hispanic x WWII | | 0.224 | | | | -0.188 | | | -0.777 | | | 0.239* | | |  |  |
|  |  | | (0.155) | | | | (0.552) | | | (1.158) | | | (0.143) | | |  |  |
|  | Asian x WWII | | -0.33 | | | | -0.0528 | | | -0.929 | | | -0.00495 | | |  |  |
|  |  | | (0.386) | | | | (0.766) | | | (1.549) | | | (0.261) | | |  |  |
|  | Native x WWII | | -0.867 | | | | 7.828 | | | -1.515 | | | -0.439 | | |  |  |
|  |  | | (1.325) | | | | (7.479) | | | (8.714) | | | (1.16) | | |  |  |
|  |  | |  | | | |  | | |  | | |  | | |  |  |
|  | Observations | | 1083000 | | | | 656000 | | | 487000 | | | 1152000 | | |  |  |
|  | Weak Instruments F | | 44.7 | | | | 0.373 | | | 10.85 | | | 45.26 | | |  |  |
|  | **Notes.** See notes to Appendix Table 1-1. The data presented in this table are approved for dissemination by the U.S. Census Disclosure Review Board (CBDRB-FY23-CES014-052, CBDRB-FY23-CES005-028, CBDRB-FY24-CES010-007, CBRDB-FY24-CES023-014, CBDRB-FY24-CES023-016). | | | | | | | | | | | | | | |  |  |

| **Appendix Table 1-7: FRD Results for Characteristics for 1969 Residence Area in Dataset for Movers and Dataset for Stayers** | | | | | | |  |
| --- | --- | --- | --- | --- | --- | --- | --- |
|  | **Table 10: Army Service Impact on Local Residence for Movers versus Stayers Within the Same Group** | | | | | | |
|  |  | (1) | (2) | (3) | |  |  |
|  | VARIABLES | Tract Wage | Tract % Same Race | County Dissimilarity Index | |  |  |
|  |  |  |  | |  |  | |
|  | **Panel 1: Dataset for Movers Who Lived in Different Counties in 1940 and 1969** | | | | | | |
|  | ARMY VETERAN COMPARISON WITH NON-ARMY MEN AMONG NON-HISPANIC WHITES | | | | | | |
|  | WWII | -342.6*** | -0.00144 | -0.00621 | |  |  |
|  |  | (75.98) | (0.00316) | (0.0041) | |  |  |
|  | DIFFERENCE-IN-DIFFERENCE BETWEEN MINORITY ARMY EFFECT AND NON-HISPANIC WHITE ARMY EFFECT | | | | | | |
|  | Black x WWII | 141.6 | -0.0229 | -0.00724 | |  |  |
|  |  | (299) | (0.0653) | (0.0288) | |  |  |
|  | Hispanic x WWII | 299.4 | -0.0479 | 0.02 | |  |  |
|  |  | (585) | (0.0558) | (0.0341) | |  |  |
|  | Asian x WWII | -1147 | -0.017 | -0.0581 | |  |  |
|  |  | (881.4) | (0.0441) | (0.0418) | |  |  |
|  | Native x WWII | -1552 | 0.0975 | -0.117 | |  |  |
|  |  | (1713) | (0.0623) | (0.0948) | |  |  |
|  | Observations | 1969000 | 1969000 | 1969000 | |  |  |
|  | Weak Instruments F | 140.1 | 140.1 | 140.1 | |  |  |
|  | **Panel 2: WWII Veteran Stayers vs. Non-veteran Stayers** | | | | | | |
|  | ARMY VETERAN COMPARISON WITH NON-ARMY MEN AMONG NON-HISPANIC WHITES | | | | | | |
|  | WWII | -314.5*** | -0.0133*** | -0.00491 | |  |  |
|  |  | (76.17) | (0.00393) | (0.00489) | |  |  |
|  | DIFFERENCE-IN-DIFFERENCE BETWEEN MINORITY ARMY EFFECT AND NON-HISPANIC WHITE ARMY EFFECT | | | | | | |
|  | Black x WWII | 292.1 | -0.0473 | -0.0519 | |  |  |
|  |  | (385.3) | (0.0964) | (0.0367) | |  |  |
|  | Hispanic x WWII | -71.72 | 0.0156 | -0.00809 | |  |  |
|  |  | (435.2) | (0.0574) | (0.0251) | |  |  |
|  | Asian x WWII | 141.1 | -0.0181 | -0.0264 | |  |  |
|  |  | (959.7) | (0.0852) | (0.0433) | |  |  |
|  | Native x WWII | -1783 | -0.268 | -0.365 | |  |  |
|  |  | (3211) | (0.349) | (0.262) | |  |  |
|  | Observations | 1156000 | 1156000 | 1156000 | |  |  |
|  | Weak Instruments F | 44.83 | 44.83 | 44.83 | |  |  |
|  |  |  |  | |  |  | |
|  | **Notes.** See notes to Appendix Table 1-1 and Appendix Table 1-4. The data presented in this table are approved for dissemination by the U.S. Census Disclosure Review Board (CBDRB-FY23-CES014-052, CBDRB-FY23-CES005-028, CBDRB-FY24-CES010-007, CBRDB-FY24-CES023-014, CBDRB-FY24-CES023-016). | | | | | | |

# **Appendix 2: Summary Statistics for Groups and Ordinary Least Squares Estimates**

**Appendix Table 2-1: Summary Statistics: Outcomes for All Groups**

| **VARIABLES** | **White** | **Black** | **Hispanic** | **Asian** | **Native American** |
| --- | --- | --- | --- | --- | --- |
| WWII Army Vet | 0.149 | 0.101 | 0.133 | 0.189 | 0.0964 |
| Filed 1040 | 0.885 | 0.626 | 0.767 | 0.789 | 0.743 |
| Any Interest | 0.587 | 0.206 | 0.347 | 0.733 | 0.33 |
| Business | 0.421 | 0.217 | 0.294 | 0.525 | 0.312 |
| log wage | 9.31 | 8.996 | 9.055 | 9.26 | 9.031 |
| log interest | 4.813 | 4.12 | 4.389 | 5.308 | 4.325 |
| log business | 5.915 | 4.749 | 5.367 | 6.361 | 5.681 |
| log adjusted gross income | 9.401 | 9.006 | 9.086 | 9.394 | 9.084 |
| Change County | 0.625 | 0.739 | 0.563 | 0.592 | 0.741 |
| Change State | 0.342 | 0.546 | 0.261 | 0.262 | 0.424 |
| Change District | 0.253 | 0.44 | 0.221 | 0.199 | 0.323 |
| Change Region | 0.202 | 0.406 | 0.152 | 0.15 | 0.263 |
| Median Wage Tract | 7116 | 5421 | 6159 | 6867 | 6366 |
| Pct Same Race Tract | 0.715 | 0.633 | 0.206 | 0.104 | 0.0395 |
| County Dissimilarity Index | 0.222 | 0.367 | 0.335 | 0.28 | 0.219 |
|  | 2877000 | 175000 | 59500 | 9000 | 4900 |

**Note:** Displays mean values of the independent and dependent variables grouped by veteran status. T-statistics are reported in the third column assuming unequal variances. White refers to non-Hispanic White men. The data presented in this table are approved for dissemination by the U.S. Census Disclosure Review Board (CBDRB-FY23-CES014-052, CBDRB-FY23-CES005-028, CBDRB-FY24-CES010-007, CBRDB-FY24-CES023-014, CBDRB-FY24-CES023-016). *** p*<*0.01, ** p*<*0.05, * p*<*0.1

| **Appendix Table 2-2: OLS Estimates for Probabilities of Filing Tax Return and Reporting Income** | | | |
| --- | --- | --- | --- |
|  | (1) | (2) | (3) |
| VARIABLES | Filed 1040 | Any Interest or Dividends | Any Business  or Farm Income |
|  |  |  |  |
| ARMY VETERAN COMPARISON WITH NON-ARMY MEN AMONG NON-HISPANIC WHITES | | | |
| WWII | 0.0247*** | 0.0113*** | 0.00505*** |
|  | (0.00171) | (0.00157) | (0.00175) |
| DIFFERENCE-IN-DIFFERENCE BETWEEN MINORITY ARMY EFFECT AND NON-HISPANIC WHITE ARMY EFFECT | | | |
| Black x WWII | 0.0830*** | -0.00855** | -0.00682 |
|  | (0.00361) | (0.00402) | (0.00434) |
| Hispanic x WWII | 0.0692*** | -0.00492 | -0.00286 |
|  | (0.0122) | (0.0131) | (0.00823) |
| Asian x WWII | 0.0908*** | 0.0317 | 0.0250* |
|  | (0.0257) | (0.0233) | (0.0128) |
| Native x WWII | 0.0132 | 0.0011 | 0.00351 |
|  | (0.0122) | (0.0231) | (0.0205) |
| DIFFERENCE BETWEEN MINORITY AND NON-HISPANIC WHITE OUTCOMES AMONG NON-ARMY MEN | | | |
| Black | -0.229*** | -0.272*** | -0.157*** |
|  | (0.00797) | (0.0081) | (0.00445) |
| Hispanic | -0.104*** | -0.175*** | -0.0971*** |
|  | (0.013) | (0.0141) | (0.00998) |
| Asian | -0.106** | 0.131** | 0.102*** |
|  | (0.0519) | (0.0514) | (0.0319) |
| Native | -0.132*** | -0.181*** | -0.0841*** |
|  | (0.0305) | (0.0234) | (0.00922) |
| Constant | 0.742*** | 0.385*** | 0.331*** |
|  | (0.0165) | (0.013) | (0.00673) |
|  |  |  |  |
| Mean | 0.861 | 0.561 | 0.407 |
| Observations | 5928000 | 3125000 | 3125000 |
| R squared | 0.044 | 0.049 | 0.013 |

**Notes.** Ordinary Least Squares Estimates from regression of outcome on World War II Service Army dummy and World War II Army Service interacted with minority group. The White coefficient is the OLS equivalent of *β_v_*, the coefficient of the Army veteran dummy in equation 1 in the text. The coefficients in the middle section are the OLS equivalents of *β_bv_* in equation 1, the coefficient on the interaction between the minority dummy and the Army veteran dummy. The coefficients in the bottom are the OLS equivalents of *β_b_* in equation 1, the coefficient on the minority group dummy variable. All regressions control for state of residence, year of birth, education, household income per capita, urban/rural residence, citizenship, indicator for having moved between 1935-1940, and trends for different birth years. The data presented in this table are approved for dissemination by the U.S. Census Disclosure Review Board (CBDRB-FY23-CES014-052, CBDRB-FY23-CES005-028, CBDRB-FY24-CES010-007, CBRDB-FY24-CES023-014, CBDRB-FY24-CES023-016). Robust standard errors in parentheses *** p*<*0.01, ** p*<*0.05, * p*<*0.1.

|  | **Table 2-3: OLS Estimates for Natural Log of Incomes for Filers Who Reported Positive Amounts** | | | | | | |
| --- | --- | --- | --- | --- | --- | --- | --- |
|  |  | (1) | (2) | (3) | (4) | | |
|  | VARIABLES | Ln(Wage &  Salary) | Ln(Interest & Dividends) | Ln(Business &  Farm) | Ln(Adjusted Gross Income) | | |
|  |  |  |  |  |  | | |
|  | ARMY VETERAN COMPARISON WITH NON-ARMY MEN AMONG NON-HISPANIC WHITES | | | | | | |
|  | WWII | 0.00711** | 0.0290*** | 0.02 | 0.0121** | | |
|  |  | (0.00345) | (0.00746) | (0.0208) | (0.00461) | | |
| DIFFERENCE-IN-DIFFERENCE BETWEEN ARMY EFFECT AND NON-HISPANIC WHITE ARMY EFFECT | | | | | | |  |
|  | Black x WWII | 0.0360*** | -0.110*** | 0.146** | 0.0341*** | | |
|  |  | (0.0063) | (0.0201) | (0.0588) | (0.00664) | | |
|  | Hispanic x WWII | 0.0147 | -0.0429 | 0.0588 | 0.00601 | | |
|  |  | (0.0135) | (0.046) | (0.0962) | (0.0181) | | |
|  | Asian x WWII | 0.0308 | -0.049 | 0.131** | 0.0507* | | |
|  |  | (0.0222) | (0.0708) | (0.0618) | (0.0276) | | |
|  | Native x WWII | 0.0234 | 0.0406 | -0.0522 | 0.00711 | | |
|  |  | (0.0412) | (0.111) | (0.152) | (0.0326) | | |
| DIFFERENCE BETWEEN MINORITY AND NON-HISPANIC WHITE OUTCOMES AMONG NON-ARMY MEN | | | | | |  |  |
|  | Black | -0.194*** | -0.496*** | -1.042*** | -0.253*** | | |
|  |  | (0.00844) | (0.0236) | (0.0446) | (0.0103) | | |
|  | Hispanic | -0.162*** | -0.298*** | -0.687*** | -0.211*** | | |
|  |  | (0.0276) | (0.0339) | (0.128) | (0.0273) | | |
|  | Asian | -0.0426** | 0.504*** | 0.0948 | -0.0111 | | |
|  |  | (0.0174) | (0.06) | (0.0601) | (0.0271) | | |
|  | Native | -0.196*** | -0.293*** | -0.311*** | -0.222*** | | |
|  |  | (0.0235) | (0.0648) | (0.0948) | (0.0232) | | |
|  | Constant | 9.003*** | 4.350*** | 5.302*** | 9.033*** | | |
|  |  | (0.0162) | (0.0375) | (0.0586) | (0.0172) | | |
|  |  |  |  |  |  | | |
|  | Mean | 9.286 | 4.795 | 5.874 | 9.372 | | |
|  | Observations | 2953000 | 1753000 | 3125000 | 3114000 | | |
|  | R squared | 0.035 | 0.027 | 0.013 | 0.067 | | |
|  | **Notes:** See notes to Appendix Table 2-2. The data presented in this table are approved for dissemination by the U.S. Census Disclosure Review Board (CBDRB-FY23-CES014-052, CBDRB-FY23-CES005-028, CBDRB-FY24-CES010-007, CBRDB-FY24-CES023-014, CBDRB-FY24-CES023-016). | | | | | | |

|  | **Table 2-4: OLS Estimates of Likelihood of Residing in Different Location in 1940 and 1969** | | | | | | |
| --- | --- | --- | --- | --- | --- | --- | --- |
|  |  | (1) | (2) | (3) | (4) | | |
|  | VARIABLES | County | State | District | Region | | |
|  |  |  |  |  |  | | |
|  | ARMY VETERAN COMPARISON WITH NON-ARMY MEN AMONG NON-HISPANIC WHITES | | | | | | |
|  | WWII | -0.0170** | -0.0115** | -0.00773* | -0.00585* | | |
|  |  | (0.00697) | (0.00508) | (0.00393) | (0.0034) | | |
| DIFFERENCE-IN-DIFFERENCE BETWEEN MINORITY ARMY EFFECT AND NON-HISPANIC WHITE ARMY EFFECT | | | | | | |  |
|  | Black x WWII | -0.0178** | -0.0179* | -0.0115 | -0.0192** | | |
|  |  | (0.00728) | (0.0095) | (0.00783) | (0.00897) | | |
|  | Hispanic x WWII | -0.0218 | -0.0111 | -0.0171** | -0.0180* | | |
|  |  | (0.0163) | (0.00799) | (0.00842) | (0.01) | | |
|  | Asian x WWII | 0.0182* | 0.0354** | 0.0275** | 0.00852 | | |
|  |  | (0.00995) | (0.0161) | (0.0124) | (0.00892) | | |
|  | Native x WWII | 0.0125 | 0.0524*** | 0.0657*** | 0.0507*** | | |
|  |  | (0.0225) | (0.0156) | (0.0181) | (0.0142) | | |
| DIFFERENCE BETWEEN MINORITY AND NON-HISPANIC WHITE OUTCOMES AMONG NON-ARMY MEN | | | | | |  |  |
|  | Black | 0.0397* | 0.155*** | 0.166*** | 0.204*** | | |
|  |  | (0.0215) | (0.0321) | (0.0308) | (0.0395) | | |
|  | Hispanic | -0.0603** | 0.0159 | 0.0257 | 0.028 | | |
|  |  | (0.0271) | (0.0332) | (0.0346) | (0.0381) | | |
|  | Asian | 0.0148 | 0.0455 | 0.0514** | 0.0517*** | | |
|  |  | (0.00979) | (0.0275) | (0.0224) | (0.018) | | |
|  | Native | 0.00897 | -0.0191 | -0.00455 | 0.00564 | | |
|  |  | (0.0199) | (0.0449) | (0.0322) | (0.0287) | | |
|  | Constant | -0.0178** | -0.0179* | -0.0115 | -0.0192** | | |
|  |  | (0.00728) | (0.0095) | (0.00783) | (0.00897) | | |
|  |  |  |  |  |  | | |
|  | Mean | 0.63 | 0.352 | 0.263 | 0.212 | | |
|  | Observations | 3125000 | 3125000 | 3125000 | 3125000 | | |
|  | R squared | 0.029 | 0.016 | 0.014 | 0.017 | | |
|  | **Note:** See notes to Appendix Table 2-2. The data presented in this table are approved for dissemination by the U.S. Census Disclosure Review Board (CBDRB-FY23-CES014-052, CBDRB-FY23-CES005-028, CBDRB-FY24-CES010-007, CBRDB-FY24-CES023-014, CBDRB-FY24-CES023-016). | | | | | | |

|  | **Appendix Table 2-5: OLS Estimates of Characteristics in Census Tract and County of 1969 Residence** | | | | | | | | |  |  |
| --- | --- | --- | --- | --- | --- | --- | --- | --- | --- | --- | --- |
|  |  | (1) | (2) | | (3) | |  |  |  |  |  |
|  | VARIABLES | Tract Wage | % Same Race | | Dissimilarity Index | |  |  |  |  |  |
|  |  |  |  |  | |  | | | |  |  |
|  | ARMY VETERAN COMPARISON WITH NON-ARMY MEN AMONG NON-HISPANIC WHITES | | | | | | | | |  |  |
|  | WWII | -25.98*** | 0.00207*** | | -0.00433*** | |  |  |  |  |  |
|  |  | (7.895) | (0.000565) | | (0.00139) | |  |  |  |  |  |
| DIFFERENCE-IN-DIFFERENCE BETWEEN MINORITY ARMY EFFECT AND NON-HISPANIC WHITE ARMY EFFECT | | | | | | | | |  |  |  |
|  | Black x WWII | 122.2*** | -0.00484 | | 0.00811** | |  |  |  |  |  |
|  |  | (19.65) | (0.00437) | | (0.00352) | |  |  |  |  |  |
|  | Hispanic x WWII | 176.3** | -0.00988 | | -0.0017 | |  |  |  |  |  |
|  |  | (79.69) | (0.0149) | | (0.00184) | |  |  |  |  |  |
|  | Asian x WWII | 130.0** | 0.00654 | | 0.00343 | |  |  |  |  |  |
|  |  | (63.2) | (0.00855) | | (0.00312) | |  |  |  |  |  |
|  | Native x WWII | 38.26 | 0.00275 | | -0.000227 | |  |  |  |  |  |
|  |  | (80.89) | (0.00416) | | (0.00572) | |  |  |  |  |  |
| DIFFERENCE BETWEEN MINORITY AND NON-HISPANIC WHITE OUTCOMES AMONG NON-ARMY MEN | | | | | | | |  |  |  |  |
|  | Black | -1511*** | -0.0659*** | | 0.0951*** | |  |  |  |  |  |
|  |  | (44.31) | (0.0117) | | (0.0112) | |  |  |  |  |  |
|  | Hispanic | -803.1*** | -0.475*** | | 0.0599*** | |  |  |  |  |  |
|  |  | (282.4) | (0.0496) | | (0.0106) | |  |  |  |  |  |
|  | Asian | -304.4*** | -0.582*** | | 0.0393*** | |  |  |  |  |  |
|  |  | (34.43) | (0.0389) | | (0.0026) | |  |  |  |  |  |
|  | Native | -533.6*** | -0.663*** | | 0.00438 | |  |  |  |  |  |
|  |  | (38.22) | (0.0133) | | (0.00643) | |  |  |  |  |  |
|  | Constant | 6649*** | 0.693*** | | 0.218*** | |  |  |  |  |  |
|  |  | (58.22) | (0.0024) | | (0.00292) | |  |  |  |  |  |
|  |  |  |  | |  | |  |  |  |  |  |
|  | Mean | 7000 | 0.698 | | 0.233 | |  |  |  |  |  |
|  | Observations | 3125000 | 3125000 | | 3125000 | |  |  |  |  |  |
|  | R squared | 0.036 | 0.262 | | 0.041 | |  |  |  |  |  |
|  | **Note:** See notes to Appendix Table 2-2. The data presented in this table are approved for dissemination by the U.S. Census Disclosure Review Board (CBDRB-FY23-CES014-052, CBDRB-FY23-CES005-028, CBDRB-FY24-CES010-007, CBRDB-FY24-CES023-014, CBDRB-FY24-CES023-016). | | | | | | | | |  |  |
|  |  | | | | | | | | |  |  |

|  | **Appendix Table 2-6: OLS Estimates of Income Reporting for Datasets of Movers and Stayers** | | | | | | |  |  |
| --- | --- | --- | --- | --- | --- | --- | --- | --- | --- |
|  |  | (1) | | (2) | | | |  |  |
|  | VARIABLES | Any Interest or Dividends | | Any Business or  Farm Income | | | |  |  |
|  |  |  |  | | |  |  |  |  |
|  | **Panel 1: Dataset for Movers Who Lived in Different Counties in 1940 and 1969** | | | | | | |  |  |
|  | ARMY VETERAN COMPARISON WITH NON-ARMY MEN AMONG NON-HISPANIC WHITES | | | | | | |  |  |
|  | WWII | 0.0139*** | | 0.00451** | | | |  |  |
|  |  | (0.00179) | | (0.00183) | | | |  |  |
| DIFFERENCE-IN-DIFFERENCE BETWEEN MINORITY ARMY EFFECT AND NON-HISPANIC WHITE ARMY EFFECT | | | | | | | |  |  |
|  | Black x WWII | -0.00168 | | -0.00515 | | | |  |  |
|  |  | (0.0049) | | (0.00585) | | | |  |  |
|  | Hispanic x WWII | 0.00915* | | 0.00285 | | | |  |  |
|  |  | (0.0054) | | (0.00729) | | | |  |  |
|  | Asian x WWII | 0.0490*** | | 0.0386*** | | | |  |  |
|  |  | (0.0157) | | (0.0116) | | | |  |  |
|  | Native x WWII | 0.00104 | | 0.0135 | | | |  |  |
|  |  | (0.0216) | | (0.0219) | | | |  |  |
|  | Observations | 1969000 | | 1969000 | | | |  |  |
|  | R squared | 0.05 | | 0.013 | | | |  |  |
| **Panel 2: Dataset for Stayers Who Lived in Same County in 1940 and 1969** | | | | | | | | | |
|  | ARMY VETERAN COMPARISON WITH NON-ARMY MEN AMONG NON-HISPANIC WHITES | | | | | | |  |  |
|  | WWII | 0.00987*** | | 0.00559** | | | |  |  |
|  |  | (0.00186) | | (0.00247) | | | |  |  |
| DIFFERENCE-IN-DIFFERENCE BETWEEN MINORITY ARMY EFFECT AND NON-HISPANIC WHITE ARMY EFFECT | | | | | | | |  |  |
|  | Black x WWII | -0.0140** | | -0.00706 | | | |  |  |
|  |  | (0.00549) | | (0.00841) | | | |  |  |
|  | Hispanic x WWII | -0.0209 | | -0.00718 | | | |  |  |
|  |  | (0.0191) | | (0.0105) | | | |  |  |
|  | Asian x WWII | 0.00362 | | 0.00509 | | | |  |  |
|  |  | (0.0319) | | (0.0215) | | | |  |  |
|  | Native x WWII | -0.00846 | | -0.0277 | | | |  |  |
|  |  | (0.0481) | | (0.0359) | | | |  |  |
|  |  |  | |  |  | | | |  |
|  | Observations | 1156000 | | 1156000 | | | |  |  |
|  | R squared | 0.046 | | 0.013 | | | |  |  |
|  | **Notes.** Ordinary Least Squares Estimates from regression of outcome on World War II Army Service dummy and World War II Army Service interacted with minority group. Top half is estimates for only movers, bottom half is estimated for only stayers. The White coefficient is the OLS equivalent of *β_v_*, the coefficient of the Army veteran dummy in equation 1 in the text. The coefficients for the minority groups are the OLS equivalent of the coefficient on the interaction between the minority dummy and the Army veteran dummy in equation 1. All regressions control for state of residence, year of birth, education, household income per capita, urban/rural residence, citizenship, indicator for having moved between 1935-1940, and trends for different birth years. The data presented in this table are approved for dissemination by the U.S. Census Disclosure Review Board (CBDRB-FY23-CES014-052, CBDRB-FY23-CES005-028, CBDRB-FY24-CES010-007, CBRDB-FY24-CES023-014, CBDRB-FY24-CES023-016). Robust standard errors in parentheses *** p*<*0.01, ** p*<*0.05, * p*<*0.1. | | | | | | |  |  |

|  | **Appendix Table 2-7: OLS Estimates of Natural Log of Income for Datasets of Movers and Stayers** | | | | | | |  |
| --- | --- | --- | --- | --- | --- | --- | --- | --- |
|  |  | (1) | (2) | (3) | (4) | | | |
|  | VARIABLES | Ln(Wage &  Salary) | Ln(Interest & Dividends) | Ln(Business &  Farm) | Ln(Adjusted Gross Income) | | | |
|  |  |  |  |  |  | | | |
|  | **Panel 1: Dataset for Movers Who Lived in Different Counties in 1940 and 1969** | | | | | | |  |
|  | ARMY VETERAN COMPARISON WITH NON-ARMY MEN AMONG NON-HISPANIC WHITES | | | | | | |  |
|  | WWII | 0.0132*** | 0.0325*** | 0.00894 | 0.0170*** | | | |
|  |  | (0.00289) | (0.0101) | (0.0231) | (0.00448) | | | |
| DIFFERENCE-IN-DIFFERENCE BETWEEN MINORITY ARMY EFFECT AND NON-HISPANIC WHITE ARMY EFFECT | | | | | |  |  |  |
|  | Black x WWII | 0.0409*** | -0.118*** | 0.146** | 0.0400*** | | | |
|  |  | (0.00553) | (0.0248) | (0.0688) | (0.00645) | | | |
|  | Hispanic x WWII | 0.0225** | 0.00319 | 0.240* | 0.0233** | | | |
|  |  | (0.00889) | (0.0508) | (0.141) | (0.00934) | | | |
|  | Asian x WWII | 0.022 | -0.0582 | 0.154** | 0.0456 | | | |
|  |  | (0.03) | (0.0634) | (0.0747) | (0.0273) | | | |
|  | Native x WWII | 0.0252 | 0.015 | 0.0861 | 0.0079 | | | |
|  |  | (0.0544) | (0.118) | (0.176) | (0.0369) | | | |
|  | Observations | 1869000 | 1098000 | 785000 | 1963000 | | | |
|  | R squared | 0.039 | 0.028 | 0.016 | 0.072 | | | |
| **Panel 2: Dataset for Stayers Who Lived in Same County in 1940 and 1969** | | | | | | |  |  |
|  | ARMY VETERAN COMPARISON WITH NON-ARMY MEN AMONG NON-HISPANIC WHITES | | | | | | |  |
|  | WWII | 0.00611* | 0.0265** | 0.0437 | 0.0127*** | | | |
|  |  | (0.00322) | (0.0116) | (0.0297) | (0.00448) | | | |
| DIFFERENCE-IN-DIFFERENCE BETWEEN MINORITY ARMY EFFECT AND NON-HISPANIC WHITE ARMY EFFECT | | | | | |  |  |  |
|  | Black x WWII | 0.0332** | -0.0738** | 0.155* | 0.0313** | | | |
|  |  | (0.0135) | (0.0288) | (0.0912) | (0.0128) | | | |
|  | Hispanic x WWII | 0.00244 | -0.0842* | -0.136** | -0.0133 | | | |
|  |  | (0.0191) | (0.045) | (0.0626) | (0.0256) | | | |
|  | Asian x WWII | 0.0383** | -0.0341 | 0.0944 | 0.0501* | | | |
|  |  | (0.0161) | (0.0938) | (0.0831) | (0.0289) | | | |
|  | Native x WWII | 0.000386 | 0.0917 | -0.46 | -0.0162 | | | |
|  |  | (0.0632) | (0.258) | (0.386) | (0.0612) | | | |
|  |  |  |  |  |  | | | |
|  | Observations | 1083000 | 656000 | 487000 | 1152000 | | | |
|  | R squared | 0.03 | 0.026 | 0.019 | 0.061 | | | |
|  | **Note:** See notes to Appendix Table 2-6. The data presented in this table are approved for dissemination by the U.S. Census Disclosure Review Board (CBDRB-FY23-CES014-052, CBDRB-FY23-CES005-028, CBDRB-FY24-CES010-007, CBRDB-FY24-CES023-014, CBDRB-FY24-CES023-016). | | | | | | |  |

|  | **Appendix Table 2-8: OLS Estimates for Characteristics of Census Tract and County for Datasets of Movers and Stayers** | | | | | | | |
| --- | --- | --- | --- | --- | --- | --- | --- | --- |
|  |  | (1) | (2) | | (3) | |  |  |
|  | VARIABLES | Tract Wage | Tract % Same Race | | County Dissimilarity Index | |  |  |
|  |  |  |  |  | |  | | |
|  | **Panel 1: Dataset for Movers Who Lived in Different Counties in 1940 and 1969** | | | | | | | |
|  | ARMY VETERAN COMPARISON WITH NON-ARMY MEN AMONG NON-HISPANIC WHITES | | | | | | | |
|  | WWII | 2.658 | 0.0179 | 0.00195*** | | -0.000777 | | |
|  |  | (8.648) | (0.0168) | (0.000435) | | (0.000477 | | |
| DIFFERENCE-IN-DIFFERENCE BETWEEN MINORITY ARMY EFFECT AND NON-HISPANIC WHITE ARMY EFFECT | | | | | | | |  |
|  | Black x WWII | 143.7*** | -0.00157 | | 0.00700*** | |  |  |
|  |  | (18.84) | (0.00452) | | (0.00261) | |  |  |
|  | Hispanic x WWII | 189.4** | -0.0192 | | -0.00287* | |  |  |
|  |  | (92.36) | (0.0121) | | (0.00144) | |  |  |
|  | Asian x WWII | 92.36 | 0.00404 | | 0.00323 | |  |  |
|  |  | (69.46) | (0.00954) | | (0.00566) | |  |  |
|  | Native x WWII | 51.34 | 0.00122 | | -0.00719 | |  |  |
|  |  | (91.72) | (0.00421) | | (0.00589) | |  |  |
|  | Observations | 1969000 | 1969000 | | 1969000 | |  |  |
|  | R squared | 0.041 | 0.271 | | 0.03 | |  |  |
|  |  |  |  |  | |  | | |
| **Panel 2: Dataset for Stayers Who Lived in Same County in 1940 and 1969** | | | | | | | | |
|  | ARMY VETERAN COMPARISON WITH NON-ARMY MEN AMONG NON-HISPANIC WHITES | | | | | | | |
|  | WWII | -29.37* | 0.00290*** | | -0.00861*** | |  |  |
|  |  | (14.64) | (0.00104) | | (0.00285) | |  |  |
| DIFFERENCE-IN-DIFFERENCE BETWEEN MINORITY ARMY EFFECT AND NON-HISPANIC WHITE ARMY EFFECT | | | | | | | |  |
|  | Black x WWII | 105.0*** | -0.0119 | | 0.00375 | |  |  |
|  |  | (27.47) | (0.0112) | | (0.00416) | |  |  |
|  | Hispanic x WWII | 171.0** | -0.0111 | | -0.00665 | |  |  |
|  |  | (81.93) | (0.0208) | | (0.0049) | |  |  |
|  | Asian x WWII | 153.1* | 0.00814 | | 0.00381 | |  |  |
|  |  | (88.22) | (0.0107) | | (0.00345) | |  |  |
|  | Native x WWII | -80.25 | 0.00735 | | 0.0141 | |  |  |
|  |  | (130.9) | (0.0133) | | (0.00963) | |  |  |
|  | Observations | 1156000 | 1156000 | | 1156000 | |  |  |
|  | R Squared | 0.036 | 0.254 | | 0.112 | |  |  |
|  |  |  |  |  | |  | | |
|  | **Note:** See notes to Appendix Table 2-6. The data presented in this table are approved for dissemination by the U.S. Census Disclosure Review Board (CBDRB-FY23-CES014-052, CBDRB-FY23-CES005-028, CBDRB-FY24-CES010-007, CBRDB-FY24-CES023-014, CBDRB-FY24-CES023-016). | | | | | | | |

|  | **Appendix Table 2-9: OLS Estimates for Filing and Reporting Income Comparing Non-Army Minorities to Non-Army Non-Hispanic White men in Dataset for Movers and Dataset for Stayers** | | | | | | | |  |
| --- | --- | --- | --- | --- | --- | --- | --- | --- | --- |
|  |  | (1) | | (2) | | | | |  |
|  | VARIABLES | Any Interest | | Any Business | | | | |  |
|  |  |  |  | | |  |  | |  |
|  | **Panel 1: Dataset for Movers Who Lived in Different Counties in 1940 and 1969** | | | | | | | |  |
|  | Constant | 0.402*** | | 0.336*** | | | | |  |
|  |  | (0.0165) | | (0.00719) | | | | |  |
|  | Black | -0.262*** | | -0.147*** | | | | |  |
|  |  | (0.00693) | | (0.00423) | | | | |  |
|  | Hispanic | -0.169*** | | -0.0885*** | | | | |  |
|  |  | (0.0111) | | (0.00556) | | | | |  |
|  | Asian | 0.119** | | 0.0923*** | | | | |  |
|  |  | (0.0514) | | (0.0315) | | | | |  |
|  | Native | -0.178*** | | -0.0826*** | | | | |  |
|  |  | (0.0236) | | (0.00649) | | | | |  |
|  | Observations | 1969000 | | 1969000 | | | | |  |
|  | R squared | 0.05 | | 0.013 | | | | |  |
| **Panel 2: Dataset for Stayers Who Lived in Same County in 1940 and 1969** | | | | | | | |  |  |
|  | Constant | 0.365*** | | 0.334*** | | | | |  |
|  |  | (0.00942) | | (0.00847) | | | | |  |
|  | Black | -0.305*** | | -0.175*** | | | | |  |
|  |  | (0.00786) | | (0.00553) | | | | |  |
|  | Hispanic | -0.183*** | | -0.114*** | | | | |  |
|  |  | (0.0202) | | (0.0157) | | | | |  |
|  | Asian | 0.147*** | | 0.116*** | | | | |  |
|  |  | (0.0494) | | (0.0297) | | | | |  |
|  | Native | -0.189*** | | -0.0868*** | | | | |  |
|  |  | (0.0309) | | (0.0191) | | | | |  |
|  |  |  | |  |  | | | | |
|  | Observations | 1156000 | | 1156000 | | | | |  |
|  | R squared | 0.046 | | 0.013 | | | | |  |
|  | **Notes.** Ordinary Least Squares Estimates from regression of outcome on World War II Service dummy and World War II Army Service interacted with minority group. Top half is estimates for only movers, bottom half is estimated for only stayers. The coefficients for the minority groups are the OLS equivalent of the coefficient on the minority dummy *β_b_* in equation 1. All regressions control for state of residence, year of birth, education, household income per capita, urban/rural residence, citizenship, indicator for having moved between 1935-1940, and trends for different birth years. The data presented in this table are approved for dissemination by the U.S. Census Disclosure Review Board (CBDRB-FY23-CES014-052, CBDRB-FY23-CES005-028, CBDRB-FY24-CES010-007, CBRDB-FY24-CES023-014, CBDRB-FY24-CES023-016). Robust standard errors in parentheses *** p*<*0.01, ** p*<*0.05, * p*<*0.1. | | | | | | | |  |

| **Appendix Table 2-10: OLS Estimates for Natural Log of Incomes Comparing Non-Army Minorities to Non-Army Non-Hispanic White men in Dataset for Movers and Dataset for Stayers** | | | | |
| --- | --- | --- | --- | --- |
|  | (1) | (2) | (3) | (4) |
| VARIABLES | Ln(Wage & Salary) | Ln(Interest & Dividends) | Ln(Business & Farm) | Ln(Adjusted Gross Income) |
|  |  |  |  |  |
| **Panel 1: Dataset for Movers Who Lived in Different Counties in 1940 and 1969** | | | | |
| Black | -0.204*** | -0.437*** | -0.962*** | -0.255*** |
|  | (0.0102) | (0.0221) | (0.0342) | (0.0121) |
| Hispanic | -0.177*** | -0.253*** | -0.676*** | -0.217*** |
|  | (0.0209) | (0.0236) | (0.15) | (0.0216) |
| Asian | -0.0325 | 0.513*** | 0.0562 | -0.0129 |
|  | (0.028) | (0.0704) | (0.0594) | (0.0329) |
| Native American | -0.192*** | -0.251*** | -0.270*** | -0.209*** |
|  | (0.0296) | (0.0573) | (0.0975) | (0.0294) |
| Constant | 9.029*** | 4.401*** | 5.429*** | 9.072*** |
|  | (0.017) | (0.0334) | (0.0741) | (0.0164) |
| Observations | 1869000 | 1098000 | 785000 | 1963000 |
| R squared | 0.039 | 0.028 | 0.016 | 0.072 |
| **Panel 2: Dataset for Stayers Who Lived in Same County in 1940 and 1969** | | | | |
| Black | -0.199*** | -0.666*** | -1.221*** | -0.276*** |
|  | (0.0154) | (0.0288) | (0.112) | (0.0181) |
| Hispanic | -0.127*** | -0.380*** | -0.762*** | -0.198*** |
|  | (0.0299) | (0.0512) | (0.118) | (0.0313) |
| Asian | -0.0622*** | 0.484*** | 0.136** | -0.0133 |
|  | (0.0148) | (0.0512) | (0.0611) | (0.0242) |
| Native American | -0.206*** | -0.410*** | -0.450*** | -0.258*** |
|  | (0.0263) | (0.0888) | (0.15) | (0.0229) |
| Constant | 8.902*** | 4.251*** | 5.139*** | 8.919*** |
|  | (0.0198) | (0.0534) | (0.0786) | (0.0241) |
|  |  |  |  |  |
| Observations | 1083000 | 656000 | 487000 | 1152000 |
| R squared | 0.03 | 0.026 | 0.019 | 0.061 |
| **Note:** See notes to Appendix Table 2-9. The data presented in this table are approved for dissemination by the U.S. Census Disclosure Review Board (CBDRB-FY23-CES014-052, CBDRB-FY23-CES005-028, CBDRB-FY24-CES010-007, CBRDB-FY24-CES023-014, CBDRB-FY24-CES023-016). | | | | |

| **Appendix Table 2-11: OLS Estimates for Characteristics of Census Tract and County of Residence Comparing Non-Army Minorities to Non-Army Non-Hispanic White men in Dataset for Movers and Dataset for Stayers** | | | | | |
| --- | --- | --- | --- | --- | --- |
|  | (1) | (2) | (3) | |  |
| VARIABLES | Tract Wage | Tract % Same Race | County Dissimilarity Index | |  |
|  |  |  |  |  | |
| **Panel 1: Non-WWII Army Veteran Movers** | | | | | |
| Black | -1648*** | -0.0704*** | 0.0948*** | |  |
|  | (44.64) | (0.0131) | (0.0112) | |  |
| Hispanic | -777.0*** | -0.520*** | 0.0512*** | |  |
|  | (244.7) | (0.0421) | (0.00448) | |  |
| Asian | -195.3*** | -0.617*** | 0.0504*** | |  |
|  | (42.08) | (0.0271) | (0.00525) | |  |
| Native American | -515.6*** | -0.677*** | 0.00432 | |  |
|  | (44.42) | (0.0111) | (0.00566) | |  |
| Constant | 6659*** | 0.695*** | 0.221*** | |  |
|  | (52.68) | (0.00242) | (0.00255) | |  |
| Observations | 1969000 | 1969000 | 1969000 | |  |
| R squared | 0.05247 | 0.2934 | 0.07174 | |  |
| **Panel 2: Non-WWII Army Veteran Stayers** | | | | | |
| Black | -1276*** | -0.0654*** | 0.0738*** | |  |
|  | (55.7) | (0.0194) | (0.0142) | |  |
| Hispanic | -713.9** | -0.412*** | 0.0594** | |  |
|  | (325.4) | (0.064) | (0.0229) | |  |
| Asian | -465.7*** | -0.528*** | 0.0220*** | |  |
|  | (59.7) | (0.0496) | (0.0072) | |  |
| Native American | -562.4*** | -0.623*** | 0.00563 | |  |
|  | (79.38) | (0.0247) | (0.0113) | |  |
| Constant | 6361*** | 0.694*** | 0.194*** | |  |
|  | (104.5) | (0.004) | (0.00634) | |  |
|  |  |  |  | |  |
| Observations | 1156000 | 1156000 | 1156000 | |  |
| R squared | 0.0445 | 0.2924 | 0.1059 | |  |
| **Note:** See note to Appendix Table 2-9. The data presented in this table are approved for dissemination by the U.S. Census Disclosure Review Board (CBDRB-FY23-CES014-052, CBDRB-FY23-CES005-028, CBDRB-FY24-CES010-007, CBRDB-FY24-CES023-014, CBDRB-FY24-CES023-016). | | | | | |

1. * Virginia Tech, Department of Economics. Email: [sbarrera@vt.edu](mailto:sbarrera@vt.edu)
2. ^†^ University of Pittsburgh, Department of Economics, and NBER. Email: [a.ferrara@pitt.edu](mailto:a.ferrara@pitt.edu)
3. ^‡^ University of Arizona, Department of Economics, and NBER. Email: [fishback@arizona.edu](mailto:fishback@arizona.edu)
4. ^§^ University of Kansas, School of Public Affairs and Administration. Email: [misty.heggeness@ku.edu](mailto:misty.heggeness@ku.edu)

   We are grateful to Melissa Thomasson and participants at the NBER Economic Impacts of World War II conference for valuable comments and suggestions. Any opinions and conclusions expressed herein are those of the authors and do not represent the views of the U.S. Census Bureau. The Census Bureau has ensured appropriate access and use of confidential data and has reviewed these results for disclosure avoidance protection (Project 7520752: CBDRB-FY23-CES014-052, CBDRB-FY23-CES005-028, CBDRB-FY24-CES010-007, CBRDB-FY24-CES023-014, CBDRB-FY24-CES023-016, CBDRB-FY25-CES010-002).
